# Molecular mechanism of abscisic acid regulating bud-break through the NnTIFY10A/B-NnABI5 module in lotus

**DOI:** 10.1093/hr/uhag125

**Published:** 2026-04-10

**Authors:** Huiyan Jiang, Fengjun Liu, Xiaojing Liu, Ping Zhou, Guangyang Liu, Qijiang Jin, Yanjie Wang, Yingchun Xu

**Affiliations:** Key Laboratory of Landscape Agriculture, Ministry of Agriculture and Rural Affairs, Key Laboratory of Flower Biology and Germplasm Development, Ministry of Agriculture and Rural Affairs, Key Laboratory of Biology of Ornamental Plants in East China, National Forestry and Grassland Administration, College of Horticulture, Nanjing Agricultural University, Nanjing 210095, China; Suzhou Academy of Agricultural Sciences, Suzhou 215000, China; Institute of Botany, Key Laboratory for Conservation and Utilization of Plant Resources, Jiangsu Province and Chinese Academy of Sciences, Jiangsu Nanjing 210014, China; Key Laboratory of Landscape Agriculture, Ministry of Agriculture and Rural Affairs, Key Laboratory of Flower Biology and Germplasm Development, Ministry of Agriculture and Rural Affairs, Key Laboratory of Biology of Ornamental Plants in East China, National Forestry and Grassland Administration, College of Horticulture, Nanjing Agricultural University, Nanjing 210095, China; Xuzhou Academy of Agricultural Sciences, Xuzhou 221000, China; Key Laboratory of Landscape Agriculture, Ministry of Agriculture and Rural Affairs, Key Laboratory of Flower Biology and Germplasm Development, Ministry of Agriculture and Rural Affairs, Key Laboratory of Biology of Ornamental Plants in East China, National Forestry and Grassland Administration, College of Horticulture, Nanjing Agricultural University, Nanjing 210095, China; Key Laboratory of Landscape Agriculture, Ministry of Agriculture and Rural Affairs, Key Laboratory of Flower Biology and Germplasm Development, Ministry of Agriculture and Rural Affairs, Key Laboratory of Biology of Ornamental Plants in East China, National Forestry and Grassland Administration, College of Horticulture, Nanjing Agricultural University, Nanjing 210095, China; Key Laboratory of Landscape Agriculture, Ministry of Agriculture and Rural Affairs, Key Laboratory of Flower Biology and Germplasm Development, Ministry of Agriculture and Rural Affairs, Key Laboratory of Biology of Ornamental Plants in East China, National Forestry and Grassland Administration, College of Horticulture, Nanjing Agricultural University, Nanjing 210095, China; Sanya Research Institute of Nanjing Agricultural University, Building 9, Wutong Industrial Park, Zhenzhou Road, Yazhou District, Sanya 572000, China

## Abstract

The apical bud-break is an important stage in the regulation of lotus (*Nelumbo nucifera* Gaertn.) flowering. Phytohormones play a key role in the development of plant buds, but the molecular mechanisms underlying the crosstalk between different phytohormone signals, especially abscisic acid (ABA) and jasmonic acid (JA), in lotus bud-break remain unclear. In this study, we found that the exogenous application of ABA inhibited the lotus apical bud-break. In addition, the expression of the gene encoding ABSCISIC ACID INSENSITIVE 5 (NnABI5), a crucial regulator of ABA signaling, was gradually downregulated during lotus apical bud-break. The transient overexpression of *NnABI5* in lotus and heterologous expression in *Arabidopsis thaliana* (L.) Heynh. demonstrated that NnABI5 negatively regulates apical bud-break and seed germination. NnABI5 interacted with the JA pathway inhibitors NnTIF[F/Y] XG 10A/B (NnTIFY10A/B), reducing the binding ability of NnABI5 to response genes *EARLY METHIONINE-LABELED 1* (*NnEM1*) and *NnEM6*. In contrast, NnTIFY10A/B positively regulates apical bud break and seed germination. Notably, exogenous application of the JA biosynthesis inhibitor DIECA alleviated the inhibitory effect of ABA. *In vitro* protein degradation assays revealed that ABA could accelerate the degradation of NnTIFY10A/B. In summary, our data reveal the crosstalk of the JA and ABA signaling pathways in lotus apical bud-break, laying a theoretical foundation for understanding the regulation of flowering in this species.

## Introduction

Upon exposure to adverse environmental conditions, some plants enter dormancy as a survival mechanism [1]. Dormancy, which dramatically slows plant growth but does not stop all biological activities [2], can be classified as bud, seed, or tuber dormancy and can be further divided into para-dormancy (known as apical dominance), endo-dormancy (inhibition by internal factors), and eco-dormancy (regulation by environmental factors) [3–5]. Bud dormancy allows perennial plants to resist harsh conditions during winter, affecting germination and flowering in the following spring [6]. Bud-break is vital for the subsequent plant growth and has attracted widespread attention.

Lotus (*Nelumbo nucifera* Gaertn.) is an economically valuable aquatic plant; its flower has high ornamental, medicinal, and edible value [7]. The perennial flowering temperate lotus variety is most commonly cultivated. It has a distinct annual growth and development period, with bud dormancy playing a major role in the regulation of flowering [8]. Jiang *et al*. found that the rate of bud-break in the spring was strongly correlated with the subsequent growth rate and flowering time of the plant [9]. Although the mechanisms of lotus apical bud-break remain unknown, Bud-break in other plants requires the crosstalk between multiple phytohormones [10]. Abscisic acid (ABA), cytokinin (CK), auxin (IAA), and gibberellin (GA) play important roles in dormancy and bud-break [11–13]. Among its many important roles in plants, ABA influences the dormancy state of perennial plant buds [14, 15]. In fruit trees, the ABA content in buds increases at the beginning of dormancy in autumn and then decreases rapidly, accompanied by changes in the expression of genes related to ABA biosynthesis and degradation [16–18]. Kim *et al*. characterized the *Arabidopsis thaliana honsu* mutant, lacking the protein phosphatase 2C (PP2C) HONSU, a key negative regulator of the ABA signaling pathway [19]. The *honsu* mutant displayed deep seed dormancy, while the *HONSU*-overexpressing plants displayed shallow seed dormancy. Many studies have reported that the ABA content, ABA biosynthesis and metabolism genes, and ABA signal transduction are also involved in the regulation of dormancy and bud break [18, 20, 21]; ABA maintains para-dormancy (axillary bud dormancy) and endo-dormancy by repressing the expression of cell cycle-related genes such as *CYCA2*/*1* and *CYCD3* [22, 23]. In peach, ABA can influence bud-break process through regulating the expression of *Myeloblastosis 52* (*PpMYB52*) [24]. However, the specific role and molecular mechanisms of ABA in the apical bud-break of lotus remain to be further investigated.

ABSCISIC ACID INSENSITIVE5 (ABI5), a bZIP family transcription factor, regulates downstream ABA signaling by binding to ABA-responsive elements (ABREs) in the promoters of its target genes, and is involved in inhibiting seed germination [25, 26]. The downregulation of *GhABI5* expression in gladiolus (*Gladiolus hybridus*) promoted bud germination [27]. In Arabidopsis, valine–glutamine motif containing proteins 18 (VQ18) and VQ26 can interact with ABI5 to inhibit its binding to the *EARLY METHIONINE-LABELED 1* (*EM1*) and *EM6* promoters, positively regulating seed germination and early seedling establishment [28]. Furthermore, numerous studies have investigated the synergistic effect of ABA and other phytohormones on plant bud germination [16, 19, 29, 30]. In sorghum (*Sorghum bicolor*), SbABI4 and SbABI5 promote seed dormancy by directly binding to the promoter region of the GA biosynthesis inhibitor gene *SbGA2ox3* (*Gibberellin 2-oxidases 3*) to inhibit GA biosynthesis [31]. In Arabidopsis, the brassinosteroid (BR) signaling pathway-related transcription factor BRI1-EMS-SUPPRESSOR 1 (BES1) directly binds to *ABI5*, thereby inhibiting the transcriptional activation of downstream genes by ABI5 and ultimately promoting seed germination [32]. Auxin Response Factors 10 (*ARF10*), and *ARF16* augment the transcriptional activity of ABI5 through direct interaction, thereby synergistically modulating the seed germination process [33–35]. In grape dormant buds, ethylene (ETH) treatment upregulates the expression of *VvA8H*-*CYP707A4*, and overexpression of this gene significantly promotes bud sprouting [36]. In *Gladiolus*, Teosinte branched1 Cincinnata Proliferating-cell factor 19 (GhTCP19) is induced by CKs and promotes bud-break by repressing the ABA biosynthesis gene *9-cis-epoxycarotenoid dioxygenase* (*GhNCED*) while enhancing the expression of the CK biosynthesis gene *isopentenyl transferases* (*GhIPT*) and signaling components *authentic response regulator* (*GhARR*) [37]. ABI5 is therefore a pivotal hub for ABA crosstalk with other phytohormones.

JA, a plant defense hormone, plays an important role in many aspects of dormancy and plant growth. In recent years, the signaling pathway of JA has been identified. When plants are subjected to stress, active JA-Ile is biosynthesized, which directly promotes the interaction between JAZ proteins and the E3 ubiquitination complex SCF^COI1^ [38–43]. This causes the Jasmonate ZIM-Domain (JAZ) proteins to be degraded by ubiquitination and activates the expression of the JA-response genes. JAZ proteins are involved in the interaction of JA with other phytohormones; for example, JAZ1/3/9 regulate the ETH signaling pathway through the interaction of their Jas domain with the ET-related transcription factors ethylene insensitive 3 (EIN3) and ethylene-insensitive 3-like 1 (EIL1) [44]. JAZ1/3/9 can also interact with DELLA proteins, core negative regulators of the GA signaling pathway, removing their inhibitory effect to activate the transcription of the GA-responsive genes [45]. MdJAZ1/2 interact with the BR-signaling protein BR INSENSITIVE1 (BRI1)-EMS-SUPPRESSOR1 (BES1)-INTERACTING MYC-LIKE PROTEIN1 (MdBIM1) to form BIM1–JAZ modules that integrate BR and JA signals to regulate plant cold tolerance in apple (*Malus domestica*) [46]. The function of JAZs in plant germination is well established [35, 47–49]; however, the mechanisms by which JAZs integrate various hormone signaling pathways to modulate bud-break remain largely unexplored.

In our previous transcriptome analysis and hormone content determination in lotus apical buds under different dormancy conditions, we determined that, with the breaking of bud, the ABA content in the apical buds decreased significantly, and the expression levels of genes related to ABA signal transduction also changed significantly [50]. Although we have found significant changes in ABA content and its pathway related genes from dormancy to bud-break, there is no direct evidence that they participate in lotus apical bud-break. In this study, we show that ABA directly promotes the expression of *NnABI5*. At the same time, and the degradation of NnTIFY10A/B further releases NnABI5, thereby activating the NnABI5–NnEM1/6 transcription cascade, which eventually inhibits lotus apical bud-break. This study provides a molecular framework for the coordination of ABA and JA in regulating lotus apical bud-break, an important process underlying flowering time in perennial plants.

## Results

### Exogenous application of ABA inhibits lotus apical bud-break

**Figure 1:**
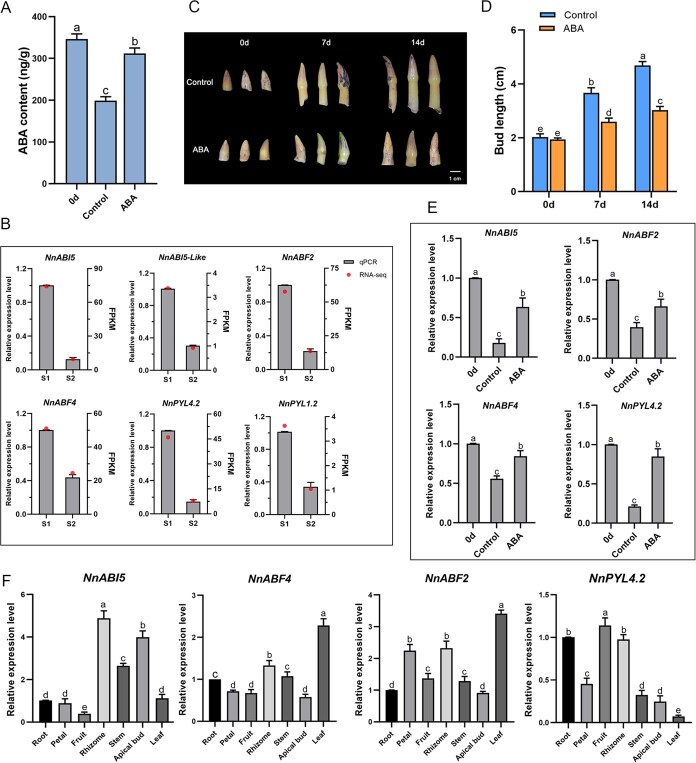
Exogenous ABA inhibits lotus apical bud-break. (A) ABA contents in the lotus apical buds after ABA treatment. Deionized water was used as the control. (B) Transcription levels of *NnABI5*, *NnABI5-like*, *NnABF2*, *NnABF4*, *NnPYL1.2*, and *NnPYL4.2* in the lotus apical buds from dormancy to bud-break. S1: dormant phase; S2: bud-break phase. Red dots represent the RNA-seq data; the gray histogram represents the RT-qPCR data. (C) Phenotypes of the lotus apical buds after the ABA treatment. The experiment was conducted at least three times with similar results. Representative images are shown. Scale bar, 1 cm. (D) Lengths of lotus apical buds after the ABA treatment. (E) Transcription levels of the ABA downstream response genes in the lotus apical buds after the ABA treatment. (F) RT-qPCR analysis of the expression levels of *NnABI5*, *NnABF2*, *NnABF4*, and *NnPYL4.2* in different organs or tissues of the lotus. *NnACTIN* was used as the internal reference gene. The data were expressed as means ± SD. Different letters indicate significantly different values, as determined using Tukey’s HSD test (*P* < 0.05).

In a previous study, we found that the dormant lotus apical buds contained a large amount of ABA, which decreased sharply with the breaking of buds (Fig. S1C). Transcriptomic analysis of lotus apical buds during the transition from dormancy to bud-break revealed that the expression levels of ABA signaling pathway-related genes, including *NnABI5*, *NnABI5-like*, *NnABF2*, *NnABF4*, *NnPYL1.2*, and *NnPYL4.2*, were significantly downregulated during bud-break process, consistent with the trend of ABA content (Figs 1B and S1A and B). These findings provide preliminary evidence for the involvement of ABA and its downstream genes in the regulation of lotus apical bud-break [50]. We therefore first explored whether ABA is involved in regulating the dormancy and breaking of lotus apical buds. Lotus rhizomes with dormant apical buds were treated with 25 μM ABA, or deionized water (as a control) to observe the breaking of apical buds. After seven days of treatment, the ABA content increased significantly after ABA treatment (Fig. 1A). Reverse transcription-quantitative PCR (RT-qPCR) results showed that ABA treatment significantly affected the expression of the differentially expressed genes identified in the transcriptomic analysis (Figs 1E and S1C). Among these, *NnABI5*, *NnABF2*, *NnABF4*, and *NnPYL4.2* exhibited the most pronounced changes in expression levels. These results demonstrate that an exogenous ABA treatment can alter the expression of ABA signal transduction genes. We observed the phenotypes of lotus dormant apical buds under the different treatments. After seven days of treatment, compared with the control, the growth of lotus buds treated with ABA was significantly inhibited. After 14 days of treatment, the inhibition effect was still evident (Fig. 1C and D). These results confirmed the inhibitory effect of ABA on lotus apical bud-break. Moreover, a tissue-specific analysis showed that the expression of *NnABI5* was significantly higher in mature rhizomes, stems, and apical buds as compared with the roots, petals, leaves, and fruit (Fig. 1F). These data further indicate that NnABI5 plays an essential role in lotus apical bud-break.

### NnABI5 negatively regulates ABA-mediated apical bud-break

A phylogenetic analysis of ABI5 homologs in various plant species showed that NnABI5 was clustered with macadamia (*Macadamia integrifolia*) MiABI5 (Fig. S2A). An amino acid sequence alignment confirmed that NnABI5 contains a conserved basic leucine zipper (bZIP) domain and belongs to the bZIP transcription factor family (Fig. S2B). A subcellular localization analysis indicated that NnABI5 is located in the nucleus (Fig. S2C).

**Figure 2:**
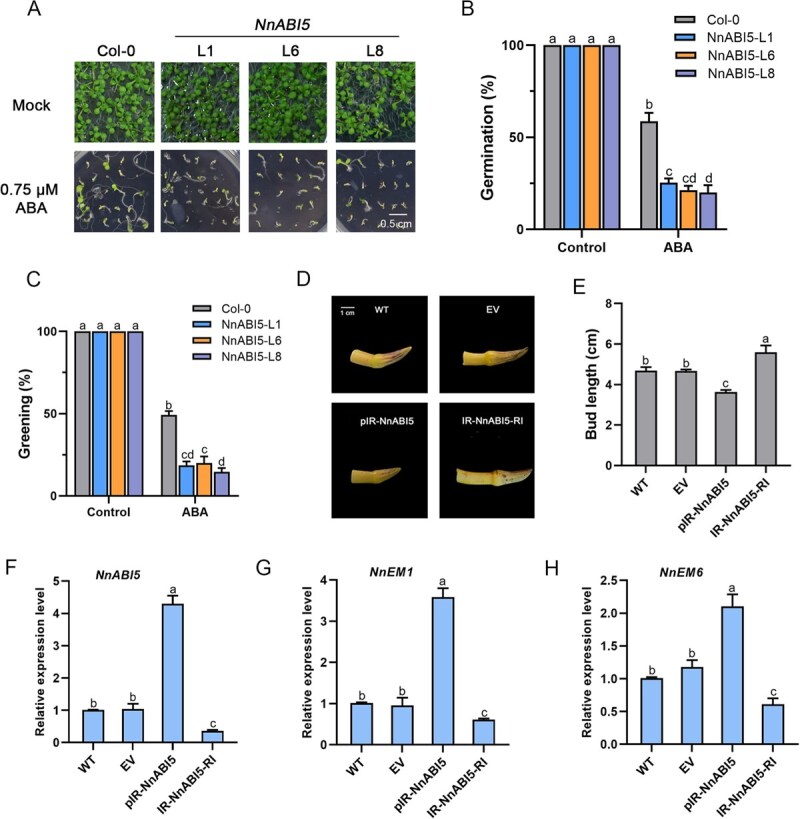
Functional verification of NnABI5. (A) Seedlings of Col-0 and Arabidopsis plants heterologously expressing *NnABI5* were grown on MS medium with or without 0.75 μM ABA. After three days of low-temperature stratification, seedlings were grown for five days under normal conditions. Representative photographs are presented. Scale bar, 0.5 cm. (B) Germination rates of Col-0 and transgenic seeds, recorded three days after the low-temperature stratification. (C) Cotyledon greening rates of Col-0 and transgenic seeds, scored five days after the low-temperature stratification. (D, E) *NnABI5*-transient overexpressed (pIR-*NnABI5*) and transient silenced (IR-*NnABI5*-RI) plants were produced using the IL-60-BS-Derived systems in the lotus dormant apical buds. Images were captured at 14 days post-infiltration. Scale bar, 1 cm. Representative phenotypes of different treatments are shown (D) and the apical bud lengths were measured (E). (F-H) Expression levels of *NnABI5* (F), *NnEM1* (G), and *NnEM6* (H) in the apical buds of transgenic lotus under different treatments, analyzed using RT-qPCR. The experiments consisted of three biological replicates and the data were expressed as mean ± SD. Using Tukey’s HSD test, different letters indicated significant difference values (*P* < 0.05).

To study the role of NnABI5 in lotus apical bud-break, we investigated the function of NnABI5 using stable heterologous expression in Arabidopsis plants. We obtained three Arabidopsis seedlings (*NnABI5* L1, L6, and L8) with stable expression of *NnABI5* (Fig. S3A). Wild-type (Col-0) and transgenic Arabidopsis seeds were planted on normal Murashige and Skoog (MS) medium, and there was no significant difference in the germination rate or cotyledon greening rate between these lines. After adding 0.75 μM ABA, the germination and cotyledon greening rates of the Col-0 seeds decreased slightly, while the *35S:NnABI5* seedlings showed more serious growth retardation, including significantly decreased germination and cotyledon greening rates (Fig. 2A–C). Next, we transiently overexpressed and silenced *NnABI5* in lotus dormant apical buds. Compared with the empty vector control, after 14 days of transient transformation, the overexpression of *NnABI5* (pIR-*NnABI5*) inhibited the apical bud growth, while the *NnABI5*-silenced (IR-*NnABI5*-RI) buds grew significantly faster (Fig. 2D and E). Our RT-qPCR analysis confirmed that the overexpression or silencing of *NnABI5* resulted in an increase or decrease in the *NnABI5* transcription level, respectively (Fig. 2F). In addition, the overexpression of *NnABI5* promoted the expression of *NnEM1* and *NnEM6*, while silencing *NnABI5* inhibited these genes (Fig. 2G and H). Collectively, these results indicate that NnABI5 enhances ABA Sensitivity in Arabidopsis seeds and inhibits lotus apical bud-break.

### *NnEM1* and *NnEM6* negatively regulate the of lotus apical bud-break

**Figure 3:**
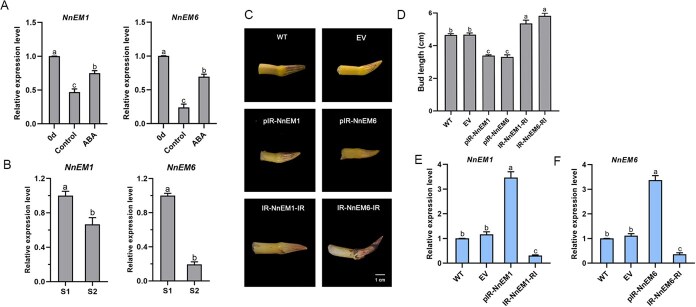
Functional verification of *NnEM1* and *NnEM6*. (A) After 14 days of ABA treatment, transcription levels of *NnEM1* and *NnEM6*. (B) Transcription levels of *NnEM1* and *NnEM6* during the dormancy-to-bud-break transition in lotus apical buds. S1: Dormant phase; S2: bud-break phase. (C and D) *NnEM1*/*6*-transient overexpressed (pIR-*NnEM1*/*6*) and transient silenced (IR-*NnEM1*/*6*-RI) plants were produced using the IL-60-BS-derived systems in the lotus dormant apical buds. Images were captured at 14 days post-infiltration. Scale bar, 1 cm. Representative phenotypes of different treatments are shown (C) and the apical bud lengths were measured (D). (E and F) Expression levels of *NnEM1* (E), and *NnEM6* (F) in the apical buds of transgenic lotus under different treatments, analyzed using RT-qPCR. The experiments consisted of three biological replicates and the data were expressed as mean ± SD. Using Tukey’s HSD test, different letters indicated significant difference values (*P* < 0.05).

*EM1* and *EM6*, members of the LEA protein family, are direct target genes of ABI5 function as downstream ABA-responsive genes, playing critical roles in maintaining seed and bud dormancy [25, 27, 51]. RT-qPCR analysis confirmed that *NnEM1* and *NnEM6* are responsive to ABA, with their expression levels progressively downregulated during lotus apical bud-break (Fig. 3A and B). To further investigate the roles of *NnEM1* and *NnEM6* in lotus apical bud-break, we transiently overexpressed and silenced *NnEM1* and *NnEM6* in lotus dormant apical buds. Compared with the empty vector control, after 14 days of transient transformation, the overexpression of *NnEM1* and *NnEM6* (pIR-*NnEM1*/*6*) inhibited the apical bud growth, while the *NnEM1* and *NnEM6*-silenced (IR-*NnEM1*/*6*-RI) buds grew significantly faster (Fig. 3C and D). Our RT-qPCR analysis confirmed that the overexpression or silencing of *NnEM1*/*6* resulted in an increase or decrease in the *NnEM1*/*6* transcription level, respectively (Fig. 3E and F).

### NnABI5 positively regulates the expression of *NnEM1* and *NnEM6*

**Figure 4:**
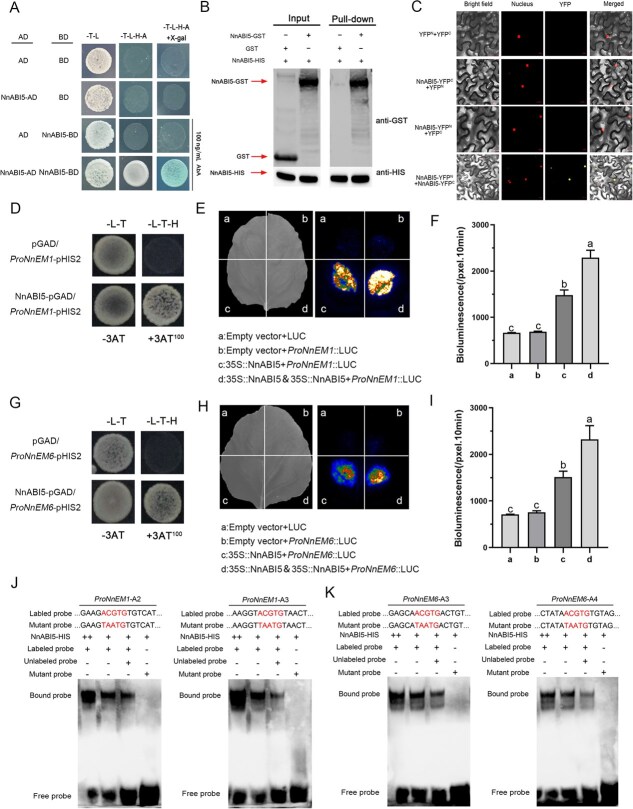
NnABI5 binds to the *NnEM1* and *NnEM6* promoters to enhance their transcription. (A) NnABI5 interacted with itself in yeast two-hybrid assays. (B) Pull-down assays were performed by co-purifying recombinant ABI5-HIS fusion proteins with ABI5-GST and the GST empty vector. Western blotting with GST antibodies showed that ABI5-GST was pulled down by ABI5-HIS. (C) NnABI5 interacted with itself in bimolecular fluorescence complementation assays. (D) Yeast one-hybrid assays revealed the binding between NnABI5 and the *NnEM1* promoter. (E) The effect of NnABI5 on the activity of the *NnEM1* promoter, determined using a dual-luciferase (LUC) reporter assay in *N. benthamiana* leaves. (a) Empty vector+LUC; (b) Empty vector+*ProNnEM1*:LUC; (c) *35S:NnABI5* + *ProNnEM1*:LUC; and (d) *35S:NnABI5* & *35S:NnABI5* + *ProNnEM1*:LUC. Representative images of *N. benthamiana* leaves are shown. (F) Quantification of the LUC bioluminescence in the *N. benthamiana* leaves (represented by panel E), performed using AndorSolis image-analysis software. The *y*-axis shows the bioluminescence per pixel over 10 min. Error bars indicate SD (*n* = 10). Different letters indicate significantly different values, determined using Tukey’s HSD test (*P* < 0.05). (G) Yeast one-hybrid assays revealed the binding between NnABI5 and the *NnEM6* promoter. (H) The effect of NnABI5 on the activity of the *NnEM6* promoter, determined using a dual-LUC reporter assay in *N. benthamiana* leaves. (a) Empty vector+LUC; (b) Empty vector+*ProNnEM6*:LUC; (c) *35S:NnABI5*+ *ProNnEM6*:LUC; and (d) *35S:NnABI5* and *35S:NnABI5* + *ProNnEM6*:LUC. Representative images of *N. benthamiana* leaves are shown. (I) Quantification of the LUC bioluminescence in the *N. benthamiana* leaves (represented by panel H), performed using AndorSolis image-analysis software. The *y*-axis shows the bioluminescence per pixel over 10 min. Error bars indicate SD (*n* = 10). Different letters indicate significantly different values, determined using Tukey’s HSD test (*P* < 0.05). (J) The competitive probes were added to verify the binding of NnABI5 to *NnEM1* (A2 and A3) motifs. (K) The competitive probes were added to verify the binding of NnABI5 to the *NnEM6* (A3 and A4) motifs. The unlabeled probe (concentration is 100 times that of the labeled probe) was used as a competitive probe. The mutant probe contains three nucleotide mutations. ‘–’ denotes that there is no corresponding protein or probe; ‘+’ indicates the presence of the corresponding protein or probe. The experiment was repeated three times with similar results. Representative images are shown here.

Through yeast two-hybrid (Y2H) experiments, we found that the Y2H yeast strain co-transformed with NnABI5-AD and NnABI5-BD could grow on the −T/−L/−H/−A selective medium containing 100 ng/ml aureobasidin A (AbA), and the colonies turned blue. Yeast strains co-transformed with NnABI5-BD and AD or NnABI5-AD and BD grew on the −T/−L selective medium, but there was no sign of growth on the −T/−L/−H/−A selective medium containing 100 ng/ml aureobasidin A (Fig. 4A). NnABI5-HIS and NnABI5-GST, NnABI5-HIS, and GST were used to perform pull-down experiments using HIS tags. Western blotting results showed that NnABI5-HIS could pull down NnABI5-GST, but NnABI5-HIS could not pull down GST (Fig. 4B). Furthermore, in bimolecular fluorescence complementation (BiFC) assays, YFP fluorescence was detected only in the nucleus of *Nicotiana benthamiana* cells co-transformed with NnABI5-YFP^N^ + NnABI5-YFP^C^, but not in cells co-transformed with NnABI5-YFP^N^ + YFP^C^ or YFP^N^ + NnABI5-YFP^C^ (Fig. 4C). Collectively, these results indicate that NnABI5 can form homodimers *in vivo* and *in vitro*.

To evaluate whether NnABI5 regulates the expression of *NnEM1* and *NnEM6* in lotus, we first isolated the promoters of *NnEM1* and *NnEM6* and ligated them to the pHIS2 vector. The interaction between NnABI5 and *NnEM1/6* was identified using yeast one-hybrid assays. As shown in Fig. 4D and G, the self-activating concentration of 3-amino-1,2,4-triazole (3-AT; a competitive inhibitor of the *HIS3* reporter gene) in the yeast strain co-transformed with *ProNnEM1/6*-pHIS2 in the −L/−T/−H selective medium was 100 mM, and the yeast strain co-transformed with NnABI5-pGADT7 and *ProNnEM1/6*-pHIS2 could grow on the −L/−T/−H selective medium containing 100 mM 3-AT. These results confirmed that NnABI5 directly binds to the *NnEM1/6* promoters in lotus. To verify the transcriptional activity of NnABI5, we performed transient expression assays in *N. benthamiana* leaves. The luminescence intensity of cells co-expressing *35S:NnABI5* and *ProNnEM1/6*:*LUCIFERASE* (*LUC*) was significantly stronger than those expressing *ProNnEM1/6*:*LUC* alone, or the empty control (Fig. 4E,F,H,I).

Subsequently, we analyzed the promoter sequences of *NnEM1* and *NnEM6* and detected several putative ABRE motifs (named A1–A4) (Supplementary Fig. S4A). We purified the NnABI5 protein and performed an electrophoretic mobility shift assay (EMSA), which revealed that NnABI5 binds to the A2 and A3 motifs in *NnEM1* and the A3 and A4 motifs in *NnEM6* (Fig. S4B). When an unlabeled probe was added as a competitor, the formation of the DNA–NnABI5 protein complex was inhibited; however, increasing the protein content of NnABI5 enabled a significant increase in the formation of the DNA–NnABI5 protein complex (Fig. 4J and K). These findings indicate that NnABI5 activates the transcription of *NnEM1* and *NnEM6*.

### NnABI5 interacts with NnTIFY10A/B *in vitro* and *in vivo*

**Figure 5:**
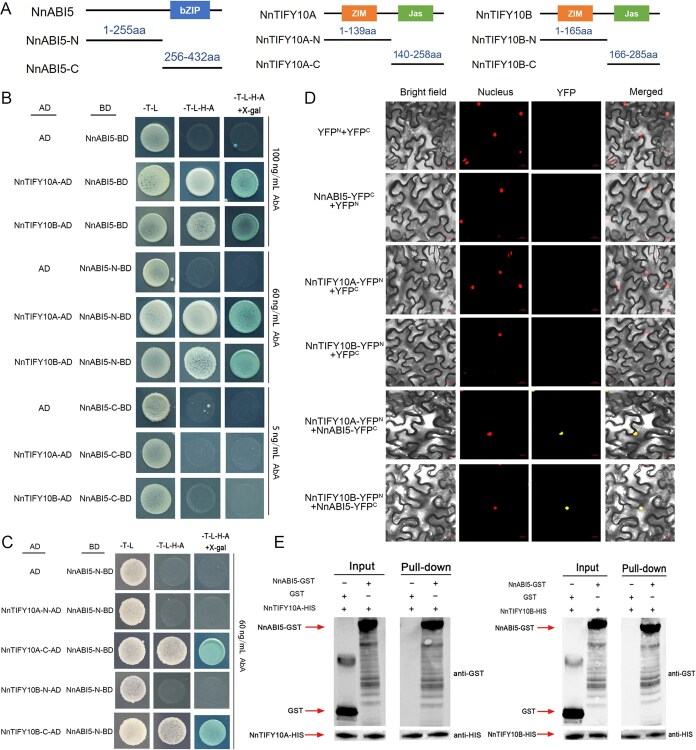
Interaction between NnABI5 and NnTIFY10A/B. (A) Diagram of the protein fragments used in the interaction analysis. The full-length NnABI5 protein was divided into C-terminal (1–255 aa) and N-terminal (256–432 aa) halves, according to the conserved domains. The full-length NnTIFY10A protein was divided into C-terminal (1–139 aa) and N-terminal (140–258 aa) halves, according to the conserved domains. The full-length NnTIFY10B protein was divided into C-terminal (1–165 aa) and N-terminal (166–285 aa) halves, according to the conserved domains. (B) Yeast two-hybrid assays of the interactions between NnTIFYA/B and different fragments of NnABI5. (C) Yeast two-hybrid assays of the interactions between NnABI5 and different fragments of NnTIFYA/B. (D) Bimolecular fluorescence complementation assays of the interaction between NnABI5 and NnTIFY10A/B *in vivo*. Scale bar, 20 μm. (E) The recombinant NnTIFY10A and NnTIFY10B fusion proteins were co-purified with the GST empty vector (Column 1) and NnABI5-GST (Column 2) for the pull-down assays. Western blotting using GST antibodies confirmed that NnABI5-GST could be pulled down by NnTIFY10A/B-HIS.

To explore the potential NnABI5 interacting factors, the lotus cDNA library was used for Y2H screening. The zinc-finger proteins NnTIFY10A and NnTIFY10B were identified as potential interacting proteins of NnABI5. A phylogenetic analysis revealed that NnTIFY10A/B are closely related to AtTIFY10A/B (Fig. S5A). A subcellular localization analysis revealed that NnTIFY10A/B are localized to the nucleus (Fig. S5B). A protein sequence comparison revealed the presence of a TIFY domain and a Jas domain in NnTIFY10A/B (Fig. S5C), belong to the Jasmonate ZIM domain (JAZ) subfamily member [52–56]. We performed Y2H assays to verify the interaction between NnABI5 and NnTIFY10A/B, observing that yeast cells containing the full-length NnABI5-AD+NnTIFY10A/B-BD sequences grew and turned blue on the −T/−L/−H/−A screening medium (Fig. 5B).

To identify the domains involved in the NnABI5–NnTIFY10A/B interaction, we divided the full-length NnABI5 protein into its C-terminal (including the bZIP domain) and N-terminal (NnABI5-N and NnABI5-C) halves. We also divided the NnTIFY10A/B proteins into N-terminal (containing the ZIM domain) and C-terminal (containing the Jas domain) halves (NnTIFY10A/B-N and NnTIFY10A/B-C) (Fig. 5A). As shown in Fig. 5C, NnABI5-N (without the bZIP domain) could interact with the NnTIFY10A/B C-terminal regions (containing the Jas domain). The results showed that the bZIP domain of NnABI5 is not required for its interaction with NnTIFY10A/B, and the Jas domain of NnTIFY10A/B is responsible for their interaction. In a BiFC assay, YFP fluorescence was detected only in the nucleus of *N. benthamiana* leaf cells co-transformed with NnABI5-YFP^N^ + NnTIFY10A-YFP^C^ or NnABI5-YFP^N^+ NnTIFY10B-YFP^C^, but was not detected in the control leaf cells (Fig. 5D). NnTIFY10A/B-HIS and NnABI5-GST, NnTIFY10A/B-HIS, and GST were used for pull-down experiments using HIS tags. NnTIFY10A/B-HIS could pull down NnABI5-GST, while NnTIFY10A/B could not pull down GST (Fig. 5E). These results indicate that NnABI5 directly interacts with NnTIFY10A/B.

### NnTIFY10A/B positively regulate lotus apical bud-break

**Figure 6:**
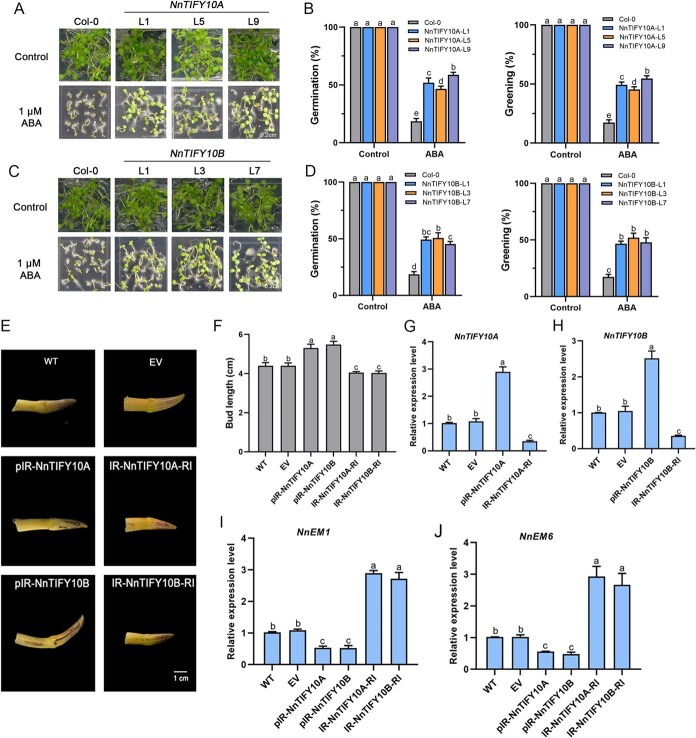
Functional verification of *NnTIFY10A* and *NnTIFY10B*. (A) Seedlings of Col-0 and Arabidopsis plants heterologously expressing *NnTIFY10A* were grown on MS medium with or without 1 μM ABA. After three days of low-temperature stratification, seedlings were grown for seven days under normal conditions. Representative photographs are presented. Scale bar, 0.5 cm. (B) Germination rates of Col-0 and transgenic seeds, recorded three days after the low-temperature stratification. Cotyledon greening rates of Col-0 and transgenic seeds, scored five days after the low-temperature stratification. (C) Seedlings of Col-0 and Arabidopsis plants heterologously expressing *NnTIFY10B* were grown on MS medium with or without 1 μM ABA. After three days of low-temperature stratification, seedlings were grown for seven days under normal conditions. Representative photographs are presented. Scale bar, 0.5 cm. (D) Germination rates of Col-0 and transgenic seeds, recorded three days after the low-temperature stratification. Cotyledon greening rates of Col-0 and transgenic seeds, scored five days after the low-temperature stratification. (E and F) *NnTIFY10A/B*-transient overexpressed (pIR-*NnTIFY10A/B*) and transient silenced (IR-*NnTIFY10A/B*-RI) plants were produced using the IL-60-BS-Derived systems in the lotus dormant apical buds. Images were captured at 14 days post-infiltration. Scale bar, 1 cm. Representative phenotypes of different treatments are shown (E) and the apical bud lengths were measured (F). (G–J) Expression levels of *NnTIFY10A* (G), *NnTIFY10B* (H), *NnEM6* (I), and *NnEM6* (J) in the apical buds of transgenic lotus under different treatments, analyzed using RT-qPCR. The experiments consisted of three biological replicates and the data were expressed as mean ± SD. Using Tukey’s HSD test, different letters indicated significant difference values (*P* < 0.05).

Because of the interaction between NnTIFY10A/B and NnABI5, we speculated that NnTIFY10A/B may play a positive role in lotus apical bud-break. We conducted two different experiments to study the roles of NnTIFY10A/B during the breaking of lotus apical buds (Fig. 6). First, we verified the function of NnTIFY10A/B in stable transgenic Arabidopsis (Fig. S3B and C). Col-0 and transgenic Arabidopsis seeds were planted on normal MS medium, and no significant differences were observed in their germination and cotyledon greening rates (Fig. 6A and C). After adding 1 μM ABA, the germination and cotyledon greening rates of the Col-0 seeds and seedlings were significantly reduced. The *35S:*NnTIFY10A and *35S:*NnTIFY10B seedlings showed less growth inhibition and higher seed germination and cotyledon greening rates than the wild type (Fig. 6B and D). Finally, we transiently overexpressed *NnTIFY10A* or *NnTIFY10B* (pIR-*NnTIFY10A/B*) in lotus dormant apical buds for 14 days, which significantly enhanced bud growth compared with that of the empty vector control (Fig. 6E and F). Silencing *NnTIFY10A* or *NnTIFY10B* (IR-*NnTIFY10A/B*-RI) had the opposite effect. Furthermore, RT-qPCR revealed that the overexpression or silencing of *NnTIFY10A/B* resulted in an increase or decrease in the *NnTIFY10A/B* transcription level, respectively, the overexpression of *NnTIFY10A* or *NnTIFY10B* inhibited the expression of *NnEM1/6* (Fig. 6I and J). These observations suggested that *NnTIFY10A/B* may play a positive role in fine-tuning the regulation of lotus apical bud-break.

### NnTIFY10A and NnTIFY10B negatively regulate the function of NnABI5

**Figure 7:**
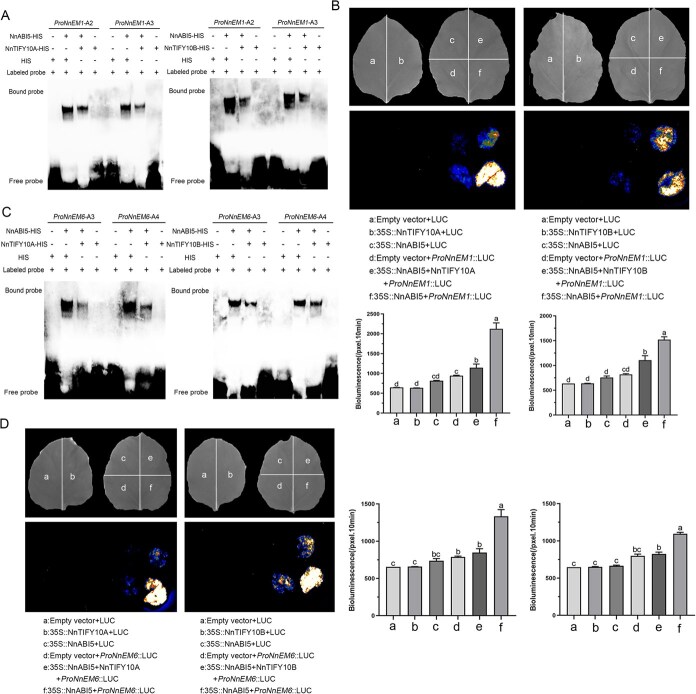
NnTIFY10A/B inhibits the activation of NnABI5 on downstream target genes. (A) and (C) Electrophoretic mobility shift assays showing that NnTIFY10A/B inhibit the binding ability of NnABI5 to the promoters of its downstream target genes *NnEM1* (A) and *NnEM6* (C). The experiment was repeated three times with similar results. Representative images are shown here. (B) and (D) The effect of NnTIFY10A/B on the ability of NnABI5 to bind to the promoters of its downstream target genes *NnEM1* (B) and *NnEM6* (D), determined using a dual-luciferase reporter assay. The quantification of the LUC bioluminescence in the *N. benthamiana* leaves was performed using AndorSolis image-analysis software. The *y*-axis shows the bioluminescence per pixel over 10 min. Error bars indicate SD (*n* = 10). (a) Empty vector+LUC; (b) *35S:NnTIFY10A* + LUC; (c) *35S:NnABI5* + LUC; (d) Empty vector+*ProNnEM1/6*:LUC; and (e) *35S:NnABI5/NnTIFY10A* + *ProNnEM1/6*:LUC; f: *35S:NnABI5* + *ProNnEM1/6*:LUC. The data were expressed as means ± SD. Different letters indicate significantly different values, determined using Tukey’s HSD test (*P* < 0.05). Representative images of the *N. benthamiana* leaves are shown.

We next tested whether NnTIFY10A/B affect the transcriptional activation function of NnABI5. In an EMSA, with the gradual addition of NnTIFY10A/B protein, the binding strength of NnABI5 to the *NnEM1/6* promoters was weakened (Fig. 7A and C). This indicates that NnTIFY10A/B reduce the binding ability of NnABI5 to the *NnEM1/6* promoters. In addition, we evaluated the effect of NnTIFY10A/B on the transcriptional activation activity of NnABI5 using a double LUC reporter gene assay. The addition of NnTIFY10A/B significantly inhibited the transcriptional activation activity of NnABI5 on *NnEM1/6* (Fig. 7B and D). These data indicate that NnTIFY10A/B reduce the transcriptional activation activity of NnABI5 on downstream target genes through direct interaction.

### ABA and JA synergistically regulate lotus apical bud-break

**Figure 8:**
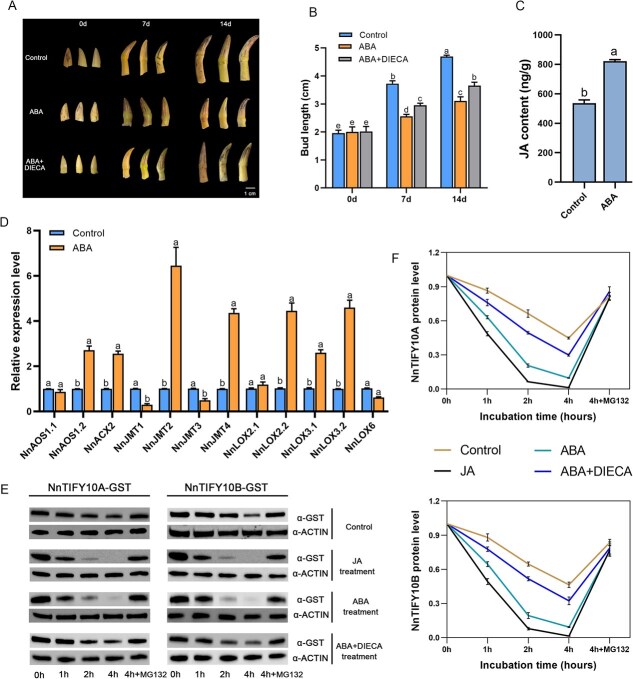
ABA regulates lotus apical bud-break partially through JA signaling pathways. (A and B) Effect of ABA and the JA inhibitor DIECA on lotus apical bud-break. Dormant lotus apical buds were treated with 25 μM ABA, 25 μM ABA+10 mM DIECA, or deionized water as a control. The experiment was carried out at least three times with similar results. Representative images are shown (A), and the apical bud lengths are quantified (B). Scale bar, 1 cm. (C) JA contents after the ABA treatment. (D) Expression levels of JA biosynthetic genes in apical buds after the ABA treatment, determined using RT-qPCR. *NnACTIN* was used as the internal reference. The data were expressed as means ± SD. Different letters indicate significantly different values, as determined using Tukey’s HSD test (*P* < 0.05). (E) JA, ABA, and ABA+DIECA treatments affected the stability of the NnTIFY10A/B proteins. The total proteins were extracted from lotus dormant apical buds treated with deionized water (control), 5 μM JA, 25 μM ABA, or 25 μM ABA+10 mM DIECA. The NnTIFY10A-GST and NnTIFY10B-GST fusion proteins were incubated with the total proteins extracted from the different treated lotus tissues. A MG132 solution was added to the incubated samples (to 100 μM) as a control. Samples were collected at the specified times. The collected samples were subjected to immunoblotting with anti-GST antibodies, and the degradation trends of the NnTIFY10A and NnTIFY10B proteins were detected. ACTIN was used as an internal reference. (F) Degradation analysis of NnTIFY10A/B-GST using the band intensity (gray value) represented in (E). The band intensity was determined using ImageJ software. The data were expressed as means ± SD.

In this study, we found that NnABI5 interacts with NnTIFY10A/B to regulate ABA-mediated lotus apical bud-break. Based on these findings, we speculated that JA, as an important plant hormone, may be involved in the ABA-mediated breaking of lotus apical buds. We treated lotus dormant apical buds with 25 μM ABA and 25 μM ABA+10 mM sodium diethyldithiocarbamate trihydrate (DIECA; a JA inhibitor) and observed the apical bud-break. After seven days of treatment, the ABA significantly inhibited the growth of the apical buds compared with the untreated buds, while the DIECA treatment alleviated the inhibitory effect (Fig. 8A and B). After 14 days of treatment, the effect was still evident. To investigate the crosstalk between ABA and JA, we measured the contents of JA and the transcript levels of the JA biosynthetic genes in the apical buds after ABA treatment. As shown in Fig. 8C and D, the JA concentration increased significantly after the ABA treatment, and five JA biosynthetic genes (*NnAOS1*, *NnACX2*, *NnJMT4*, *NnLOX2.2*, and *NnLOX3*) also showed significantly increased expression levels. *In vitro* protein degradation experiments showed that the JA and ABA treatments promote the degradation of the NnTIFY10A/B proteins, with the degradation-promoting effect of JA more obvious than that of ABA. Compared with the ABA treatment alone, the ABA+DIECA treatment significantly inhibited the degradation of the NnTIFY10A/B proteins. Moreover, treatment with the proteasome inhibitor MG132 inhibited this degradation (Fig. 8E and F). In summary, these findings suggest that ABA may regulate lotus apical bud-break partially through JA signaling pathways.

## Discussion

Bud dormancy is a self-protection mechanism used by perennial plants to survive harsh environmental conditions, and is of great significance to the agricultural industry. ABA plays a crucial role in the life cycle of plants, including seed dormancy, germination, stomatal movement, and fruit development, as well as in biotic and abiotic stresses [55–57]. In this study, we found that exogenous ABA treatments significantly inhibited the lotus apical bud-break (Fig. 1). This is consistent with previous studies in lily (*Lilium davidii* var*. unicolor*) [58], pear (*Pyrus pyrifolia*) [18], gladiolus [27], and grape (*Vitis vinifera*) [15].

The bZIP family transcription factor ABI5 is a key positive regulator of ABA signaling and plays an important role in biological processes such as seed germination and dormancy, plant growth and development, responses to stress, and the crosstalk between various plant hormones [59–62]. Here, we isolated a bZIP transcription factor, NnABI5, from lotus. Our RNA-seq data showed that the expression level of *NnABI5* was significantly downregulated with the breaking of lotus apical bud (Fig. 1), a trend consistent with ABA-responsive genes in other perennial plants, suggesting that NnABI5 plays a role in lotus apical bud-break. This hypothesis was confirmed by the inhibition of lotus apical bud-break following the transient overexpression of *NnABI5* and the inhibition of germination in Arabidopsis seeds heterologously expressing *NnABI5* (Fig. 2). Together, these results demonstrate that ABI5 has a conserved inhibitory function in both bud-break and seed germination process.

The *EM1* and *EM6* genes play crucial roles in seed germination [21]. ABI5 directly binds to the promoters of *EM1* and *EM6* genes, participating in seed germination by regulating the transcription of target genes [25, 26]. Wu *et al*. demonstrated that silencing GhABI5 downregulates the expression of ABA downstream genes (e.g. *GhLEA* and *GhRD29B*), thereby effectively promoting the corm sprouting process in *Gladiolus* [27]. In the present study, transient infection assays confirmed the inhibitory effects of *NnEM1*/*6* on the lotus apical bud-break (Fig. 3). Through yeast one-hybrid, EMSA, and LUC assays, we established that NnABI5 binds to the downstream *NnEM1*/*6* promoters and activates their expression in lotus (Fig. 4). This suggests that, similar to mechanisms observed in seeds, NnABI5 likely establishes and maintains dormancy in lotus apical buds by directly activating the expression of germination-inhibiting genes such as *NnEM1*/*6*. Furthermore, NnABI5 can form homodimers (Fig. 4), this indicates that ABI5 may possesses various mechanisms to activate downstream ABA responses. This finding implies a potentially complex hierarchical regulation of its transcriptional activity, warrants further investigation.

ABI5 serves as a pivotal node in the activation of ABA and other plant hormone signaling pathways, thereby synergistically regulating plant growth and development [63–66]. The key inhibitor of the BR signal transduction pathway, BRASSINOSTEROID INSENSITIVE 2 (BIN2), interacts with ABI5 and activates its transcriptional activation activity to positively regulate ABA signaling [64]. RGA-LIKE 2 (RGL2; a negative regulator of the GA signaling pathway) and ABI5 are involved in the interaction of ABA and GA in the regulation of seed germination [67]. Here, we identified the JAZ proteins NnTIFY10A/B as the interaction partners of NnABI5 (Fig. 4). JAZ proteins alone do not have formal functions, but are involved in regulating JA and other signal transduction pathways by interacting with different domains of specific proteins to form different complexes [68]. Our study showed that NnTIFY10A or NnTIFY10B can negatively regulate NnABI5 activation of the downstream target genes (Fig. 7). We speculate that JAZ proteins likely achieve this repression by directly binding to ABI5 and occluding its transcription activation domain. This mechanism shows high consistency with findings in other species; for instance, in apple, MdJAZ1 and MdJAZ2 proteins have been reported to interact with and inhibit the activity of transcription activators MdBBX37 and MdBIM1, thereby attenuating their transactivation of the downstream *MdCBF* genes [46, 69]. Our physiological assays further confirmed that transiently overexpressed *NnTIFY10A* or *NnTIFY10B* in lotus to demonstrate their positive regulation of lotus apical bud-break, and heterologously expressed them in Arabidopsis to reveal their positive influence on seed germination (Fig. 6). JAZ proteins interact with ABI5 to regulate seed germination in wheat (*Triticum aestivum*) [47]. In Arabidopsis, the JAZ proteins regulate the function of ABI5 to integrate the JA and ABA signals during seed germination and post-germination growth [48]. Our work has confirmed that the ABI5-JAZ module plays a general role in plant germination, and relevant studies have shown that ABI5-JAZ also functions as a core module in other aspects [70]. Our discovery provides a new perspective for understanding the diversity and specificity of hormone crosstalk in different plant species.

Although it has been extensively documented that JA promotes the ABA-mediated delay in seed germination [47, 48, 71], the synergistic relationship between the two pathways is yet to be fully elucidated. Previous measurements of hormone levels in apical buds from dormancy to bud-break revealed a significant decrease in JA content. Transcriptomic analysis showed that NnTIFYs, negative regulators of JA, were up-regulated [50]. In the present study, the exogenous application of DIECA (a JA inhibitor) significantly alleviated the inhibition of lotus apical bud-break under an ABA treatment (Fig. 8A and B). We therefore speculate that the inhibitory effect of ABA is partially mediated through the JA pathway. Recent studies on seed germination in Arabidopsis have shown that exogenous ABA can regulate *AOC* transcription via the SAPK10–bZIP72 module, increase the concentration of JA in seeds, and synergistically inhibit seed germination [72]. Our findings indicate that the ABA treatment promoted the expression of the JA biosynthetic genes *AOS1*, *ACX2*, *JMT4*, *LOX2.2*, and *LOX3*, further promoting JA accumulation (Fig. 8C and D). Although we can hypothesize that ABA may specifically promote JA accumulation through the promotion of these biosynthetic genes based on our quantitative results, the specific molecular mechanism still requires further research to confirm or disprove this theory. Additionally, other studies have reported mechanisms by which ABA regulates JA biosynthesis; for instance, Liu et al. suggested that the ABA-response gene *OsbZIP82* may positively regulate the JA concentration by acting directly on JA signaling and metabolic pathway genes rather than on JA biosynthetic genes in rice [73]. In Arabidopsis chloroplasts, ABA may enhance the biosynthesis of JA by regulating the expression of plastid phospholipase genes such as *PLIP2* and *PLIP3* [74]. Collectively, these insights provide diverse avenues for future research endeavors. In summary, we conclude that the ultimate inhibitory effect of ABA on lotus apical bud-break likely arises from a dual inhibitory action involving both direct effects of ABA and its induced accumulation of JA coupled with subsequent signal transduction. Moreover, building on relevant research, future studies could further investigate the direct effects of exogenous JA application on lotus apical bud-break, as well as the regulatory role of the JA biosynthesis inhibitor DIECA on endogenous JA levels. This would help to more comprehensively elucidate the molecular network through which ABA and JA synergistically regulate bud dormancy and breaking.

**Figure 9:**
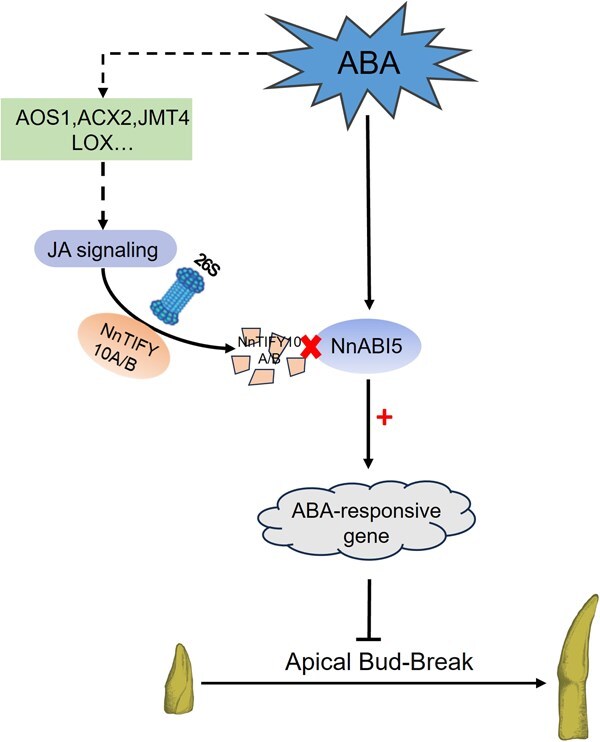
Dynamic model of NnTIFY10A/B-NnABI5 regulates lotus apical bud-break.

In this study, we constructed a dynamic model that reveals the molecular mechanism by which the NnTIFY10A/B-NnABI5 complex regulates ABA-mediated lotus apical bud-break (Fig. 9). In the presence of ABA, NnTIFY10A/B are degraded through the 26S-proteasome pathway [75, 76], releasing NnABI5 and activating the NnABI5–NnEM1/6 signaling cascade. Consequently, the lotus apical bud-break is inhibited. When the ABA level in the plant is low, NnTIFY10A/B protein levels are high, which reduces the ability of NnABI5 to bind to its downstream target genes *NnEM1/6*, thereby promoting lotus apical bud-break.

In the present of ABA, two synergistic mechanisms collectively inhibit the lotus apical bud-break. Firstly, ABA directly upregulates the transcription of *NnABI5* and *NnEM1*/*6*, then inhibiting lotus apical bud-break; On the other hand, ABA modulates the protein degradation of NnTIFY10A/B, thereby releasing NnABI5 and activating the NnABI5-NnEM1/6 signaling cascade. These dual regulatory pathways ultimately suppress ABA-mediated apical bud-break in lotus. In the model, the arrows indicate a positive relationship, while the short vertical line indicates a negative relationship.

## Materials and methods

### Plant materials and culture conditions

The lotus cultivar *N. nucifera* ‘Aijiangnan’ was used as the experimental material in this study. It was planted in the Bai Ma experimental base at Nanjing Agricultural University (31°95′N, 118°85′E), Nanjing, China, and grown in the natural environment. According to previous studies on the definition of lotus dormancy time [50, 77], the apical buds of lotus were harvested in February 2023–2024 (close to the ecological dormancy period) and used for the hormone treatments and transient transformation experiments. Similar-sized lotus rhizomes with 1.5 ± 0.5 cm apical buds were cultured in an artificial climate chamber (22 ± 0.5°C, 16 h light/8 h dark). The materials for the plant tissue specificity experiments were taken from petals, leaves, and stems that bloomed in May 2022, and fruits, roots, apical buds, and rhizomes that entered dormancy in October of the same year.

*Arabidopsis thaliana* Columbia-0 (Col-0) plants and *N. benthamiana* seedlings were grown in an incubator at 22°C with a 16 h light/8 h dark cycle.

### Effects of ABA and JA treatment on dormancy

The lotus rhizomes with apical buds collected in February were soaked in deionized water containing 25 μM ABA [27, 58], or 10 mM DIECA [78], or in deionized water as an untreated control. The samples were then placed in an artificial climate chamber (22 ± 0.5°C, 16 h light/8 h dark). The length of the apical buds was measured and photographed after seven and 14 days, and samples were taken after seven and 14 days for subsequent testing. Each treatment had at least six apical buds. The experiment was independently repeated three times.

### Phylogenetic tree and sequence alignment

The NnABI5 protein sequence and ABI5 protein sequences of 20 different species were downloaded from the National Center for Biotechnology Information (NCBI) database. The multiple sequence alignment was performed using the ClustalW tool, employing the neighbor-joining method with a bootstrap replication count of 1000 in MEGA 7.0 software (http://www.megasoftware.net), to analyze the evolutionary relationship of the ABI5 proteins [79]. The ABI5 protein sequences of 10 species were compared using DNAMAN software (https://www.lynnon.com).

### Plasmid construction and transient transformation

Based on the improved tomato yellow leaf curl virus (TYLCV)-based geminivirus vector system (IL-60-BS/IR), transient transformation experiments were conducted for gene overexpression or silencing in lotus, as previously described [80–83]. The overexpression vector (pIR-X) or silencing vector (IR-X-RI) and IL-60-1 plasmid were injected into lotus rhizomes at a ratio of 1:1 (800 ng/100 μl), once every three days. After 14 days from the first transformation, the plant phenotype was observed, and the gene expression levels were assessed. Five plants were used for each transfection experiment, for each gene transfection experiment, a total of twenty apical buds were injected for treatment. The experiment was independently repeated three times. The primers used are listed in Table S1.

### Construction of the expression vector and acquisition of transgenic *A. Thaliana*

The full-length coding sequences (CDS) of *NnABI5* and *NnTIFY10A/B* were cloned into the pENTR-D-TOPO vector (Thermo Fisher Scientific, Waltham, MA, USA) and then recombined into the binary vector pFAST-R05. Transgenic *A. thaliana* plants were obtained using the floral dip transformation method [50, 84]. The primers used are listed in Table S1.

### RNA-seq analysis

Transcriptome sequencing was performed by Gene Denovo Biotechnology Co. (Guangzhou, China), where cDNA libraries were constructed and sequenced on an Illumina Novaseq 6000 [50]. Transcriptome data has been deposited in CNCB, the accession number is PRJCA019379.

### Total RNA extraction and RT-qPCR analysis

The Plant Total RNA Isolation Kit (RC411; Vazyme, Nanjing, China) was used to extract RNA from different tissues of lotus, according to the manufacturer’s recommended protocol. RNA was then reverse-transcribed using HiScript II Q RT SuperMix for qPCR (+ gDNA wiper) (R223; Vazyme). qPCR analyses were performed using SYBR Green PCR (Q311; Vazyme) with a CFX96 Real-Time PCR Detection system (Bio-Rad Laboratories, Hercules, CA, USA). All quantitative primer sequences in this experiment are shown in Table S1. *NnACTIN* was used as the internal reference gene [85]. The relative expression levels of the genes of interest were determined using the 2^−ΔΔCt^ method [86].

### Subcellular localization

The CDSs of *NnABI5* and *NnTIFY10A/B* were amplified and cloned into the pRI101-AN vector, which contains a GFP tag, under the regulation of the cauliflower mosaic virus *35S* (CaMV35S) promoter. The recombinant vectors 35S:NnABI5-GFP and 35S:NnTIFY10A/B-GFP were transformed into *Agrobacterium tumefaciens* GV3101 strain. The transformed strain was then infiltrated into *N. benthamiana* epidermal leaf cells. The RFP (detection wavelength: 481–549 nm) and GFP (detection wavelength: 562–650 nm) fluorescence signals in *N. benthamiana* cells were visualized using a confocal laser scanning microscope (LSM 800; Carl Zeiss, Oberkochen, Germany). An empty vector was used as the control. The primers used for the amplification are listed in Table S1.

### Y2H screening and assays

The CDS of *NnABI5* was cloned into the pGBDT7 bait vector, the cDNA library is a self-stored resource in the laboratory and was prepared using mixed lotus flower buds as the material, with the specific methodologies and tissue selection procedures following the methods described by Li *et al*. [83, 87]. The lotus Y2H library screening experiment was carried out using a yeast transformation system (Takara Bio, Kusatsu, Japan). The full-length CDS of *NnTIFY10A/B* was also inserted into the pGADT7 vector. Different combinations of recombinant plasmids were co-transformed into the yeast strain ‘Y2H Gold’ using polyethylene glycol (PEG)-mediated genetic transformation to observe the growth of yeast cells on SD/−Trp/−Leu (−T/−L) and SD/−Trp/−Leu/−His/−Ade (−T/−L/−H/−A) media containing different concentrations of aureobasidin A. The primers used are listed in Table S1.

### Pull-down and BiFC assays

The pull-down and BiFC assays were performed to further determine the interaction between the NnABI5 and NnTIFY10A/B proteins. For the pull-down assays, *NnTIFY10A/B* were recombined into the expression vector pET32a containing the HIS tag, and *NnABI5* was recombined into the expression vector pGEX4T-1 containing the glutathione S-transferase (GST) tag. The recombinant plasmids were transformed into *Escherichia coli* BL21 (DE3) and induced to express the NnABI5-GST and NnTIFY10A/B proteins. Subsequently, pull-down assays were performed using the HIS-Tagged Protein Purification Kit (CWbiotech, Beijing, China). The elution solutions were analyzed in immunoblots using anti-HIS and anti-GST antibodies (Abmart, Shanghai, China). The specific methods used were described previously [88].

For the BiFC assays, the CDS of *NnABI5* and *NnTIFY10A/B* (without the termination codon) were cloned and ligated into the pSPYCE-YFP and pSPYNE-YFP vectors, respectively. The recombinant vectors were transformed into *A. tumefaciens* GV3101 strain cells, which were then injected into the *N. benthamiana* leaves. The YFP fluorescence signals were observed with an epifluorescence microscope (LSM800; Carl Zeiss) after a dark treatment for 12 h and normal culture for two days. The primers used are listed in Table S1.

### Yeast one-hybrid assays

The CDS of *NnABI5* and the promoter sequences of *NnEM1/6* were recombined into the pGADT7 and pHIS2 vectors, respectively. The resulting recombinant plasmid was transformed into the Y187 yeast strain and plated on SD/−Leu/−Trp/−His and SD/−Leu/−Trp/−His+100 mM 3-AT media. The interaction was determined by observing the growth of yeast in a 28°C incubator. The primers used are listed in Table S1.

### Dual-LUC assays

The CDSs of *NnABI5* and *NnTIFY10A/B* were cloned into the pGreenII 62-SK vector to produce effector constructs. Promoter fragments of *NnEM1/6* were cloned into the pGreenII 0800-LUC vector to generate reporter constructs (Fig. S6). Next, various combinations of plasmids were transformed into *N. benthamiana* cells using the *Agrobacterium*-mediated transformation method. After 2–3 days, LUC images were observed using a live-cell imaging apparatus (PIXIS 1024B; Teledyne Princeton Instruments, Trenton, NJ, USA). The LUC activity was measured using Andor Solis image-analysis software (Andor Technology, Belfast, UK). The primers used are listed in Table S1.

### EMSAs

The EMSAs were conducted with the LightShift TM Chemiluminescent EMSA kit (Thermo Fisher Scientific), as described previously [89]. The biotin probes (Table S2) were designed according to the ABRE element on the promoter sequences of *NnEM1* and *NnEM6* and synthesized by Sangon Biotech (Shanghai, China). NnABI5-HIS or NnTIFY10A/B-HIS purified proteins and probes (biotin-labeled, unlabeled, and mutant probes) were mixed in binding buffer and incubated at room temperature for 30 min. The separation between free and bound probes was elucidated following development through acrylamide gel electrophoresis and subsequent membrane transfer. The unlabeled probes were used as a competitor, and the mutant probes were used as a control.

### Protein degradation assays

The lotus rhizomes with apical buds collected in February were soaked in deionized water containing 25 μM ABA [27, 58], 5 μM MeJA [78], or 25 μM ABA+10 mM DIECA [78] for 24 hours, with deionized water alone used as an untreated control. The total proteins were extracted from different treated lotus tissues, as described previously [90, 91]. The total proteins were incubated with purified NnABI5-GST or NnTIFY10A/B-GST proteins. The samples were collected and boiled at different time points, and the subsequent analysis was carried out by immunoblotting. The antibodies used for detection included anti-GFP (1:5000; Abmart), anti-ACTIN (1:5000; Abmart), and anti-mouse IgG (1:2000; Abmart).

### Determination of the JA and ABA contents

The ABA and JA contents in the lotus tissues were measured using a plant enzyme-linked immunosorbent assay (ELISA) kit (F4585-B; F7890-B; Meimian, Shanghai, China).

### Statistical analysis

Tukey’s honestly significant difference (HSD) tests were performed using SPSS 22.0 (IBM, Armonk, NY, USA) to detect significant differences in the data. Different lowercase letters were used to denote significant differences (*P* < 0.05) on the graphs. The histograms were generated using GraphPad Prism 8.0 software (GraphPad, San Diego, CA, USA). All experiments were repeated at least three times.

### Accession numbers

Sequence data from this article can be found in the NCBI database under the following accession numbers: *NnABI5* (LOC104588466), *NnTIFY10A* (LOC104609287), *NnTIFY10B* (LOC104600505), *NnEM1* (LOC104602566), *NnEM6* (LOC104594933), *AtABI5* (AT2G36270), *AtEM1* (AT3G51810), and *AtEM6* (AT2G40170).

## Supplementary Material

Web_Material_uhag125

## Data Availability

All data supporting the findings of this study are available within the paper and within its supplementary data published online.
