# Supplementary material for: Molecular mechanism of abscisic acid regulating bud-break through the NnTIFY10A/B-NnABI5 module in lotus
Source: Hortic Res. 2026 Apr 10;13(8):uhag125. doi: 10.1093/hr/uhag125 (PMC13404126; doi:10.1093/hr/uhag125)
Supplement: Web_Material_uhag125 [file web_material_uhag125.zip › Supplementary figures.docx]

Supplementary Data
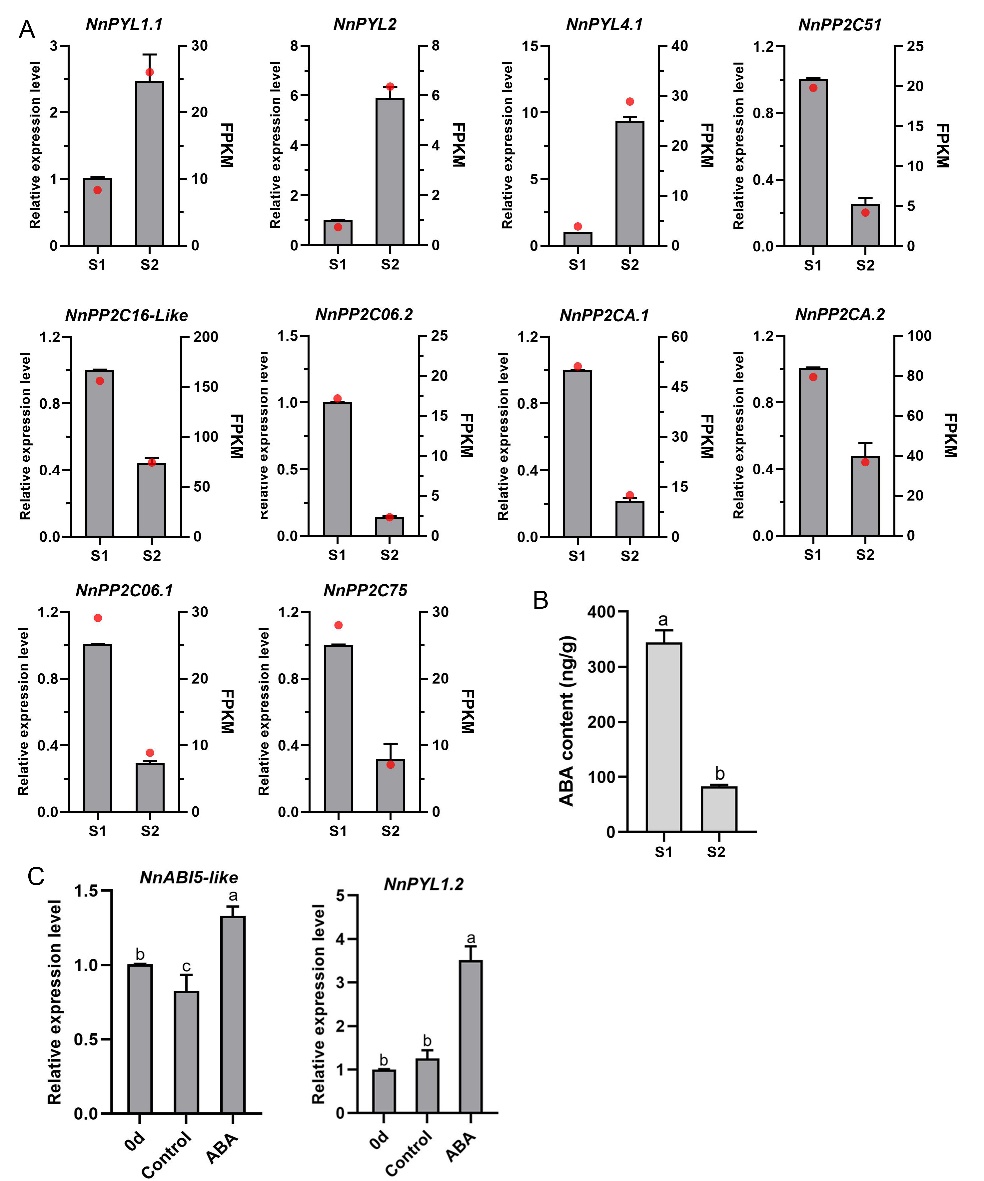


**Figure S1.** Transcription levels of the downstream ABA response genes.

(A) Transcription level of *NnPYL1.1*, *NnPYL2*, *NnPYL4.1*, *NnPP2C16-like*, *NnPP2C51*, *NnPP2C06.1*, *NnPP2C06.2*, *NnPP2CA.1*, *NnPP2CA.2*, and *NnPP2C75* in the lotus apical buds from dormancy to dormancy release. Red dots represent the RNA-seq data; the gray histogram represents the RT-qPCR data.

(B) ABA contents in the lotus apical buds from dormancy to dormancy break.

(C) Transcription levels of *NnABI5-like* and *NnPYL1.1* in lotus apical buds after the ABA and fluridone treatments. The data were expressed as means ± SD. Different letters indicate significantly different values, determined using Tukey’s HSD test (*P* < 0.05).


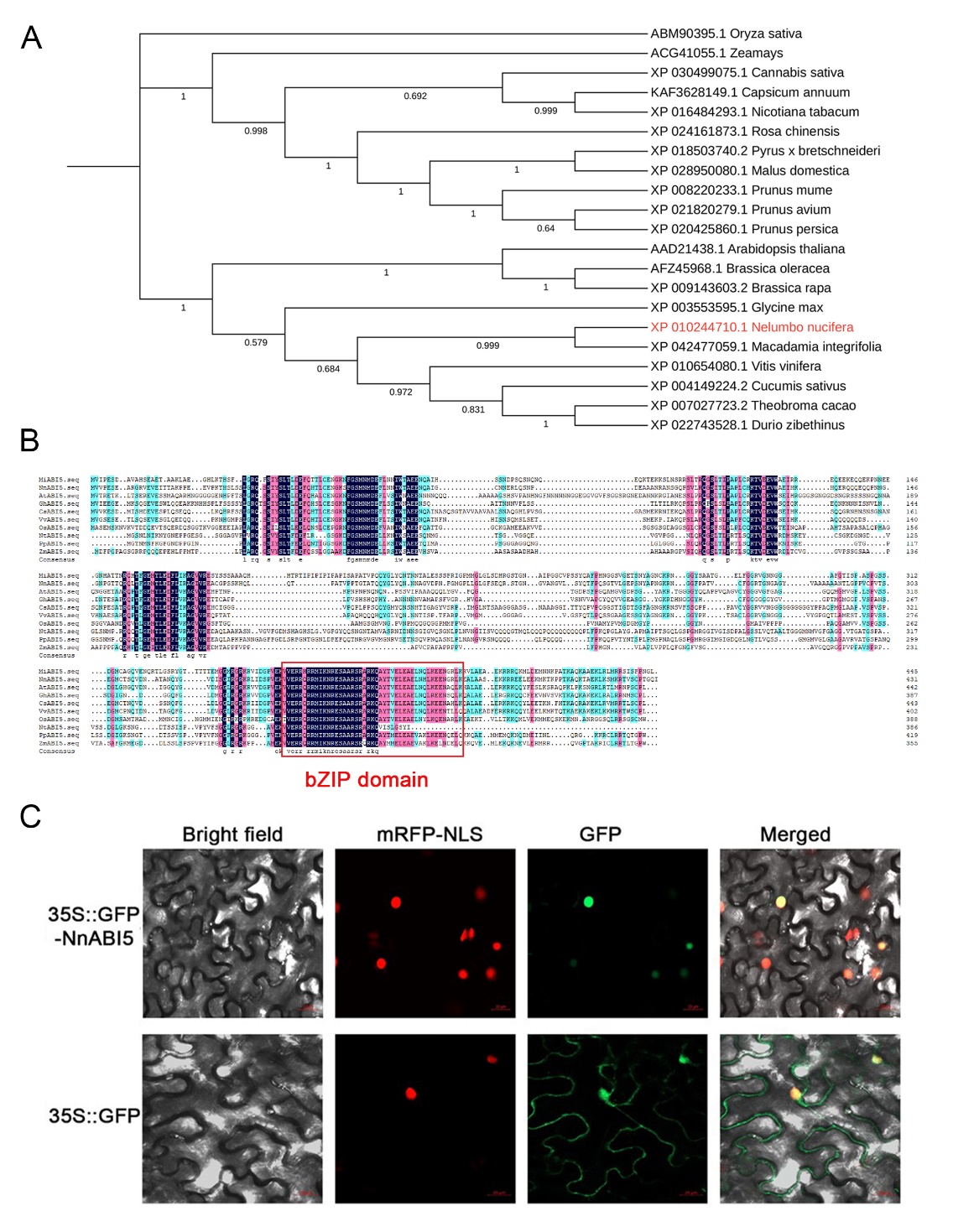


**Figure S2.** Bioinformatics analysis of NnABI5.

(A) A Phylogenetic tree analysis of NnABI5 in different species.

(B) An Amino acid sequence alignment of NnABI5 in different species, and the red box represents the bZIP domain.

(C) Subcellular localization of NnABI5 in *N. benthamiana* leaves based on NnABI5-GFP fusion. Scale bar, 20 µm.

**
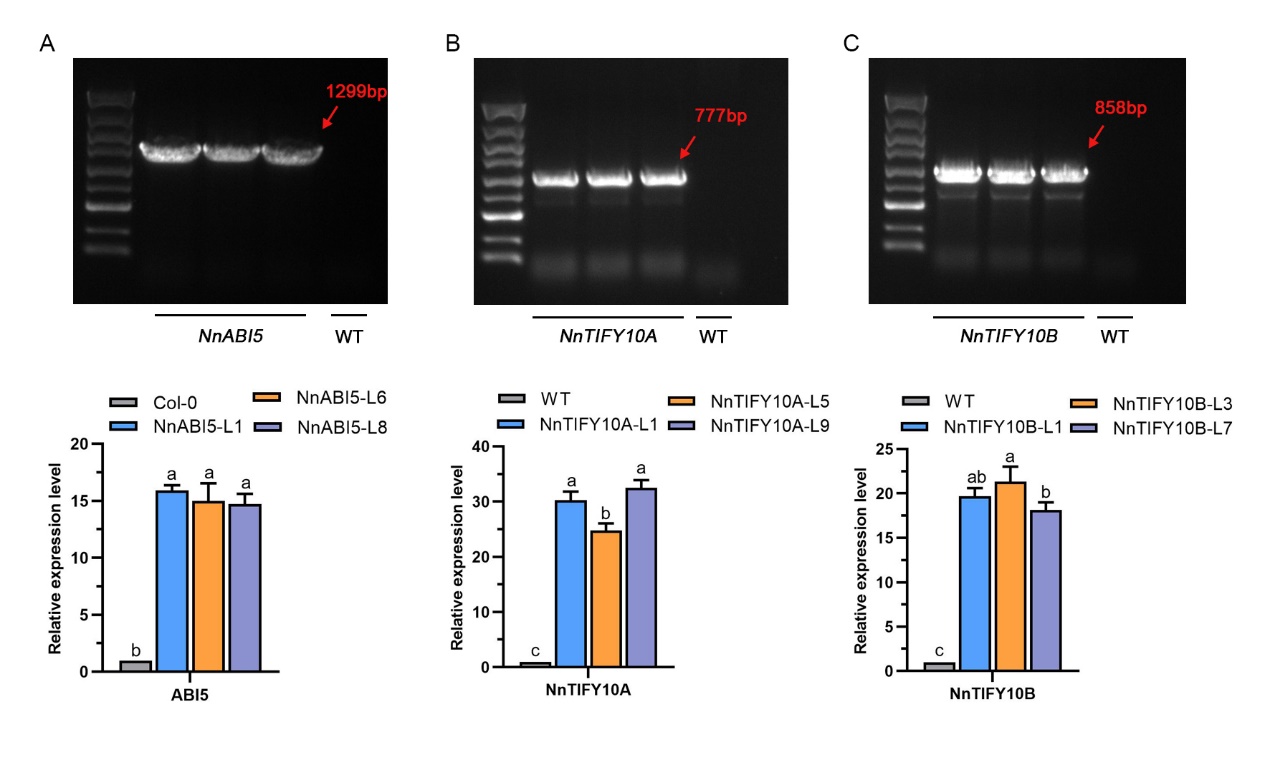
**

**Figure S3.** Transgene presence in Arabidopsis lines expressing *NnABI5*/*NnTIFY10A* /*NnTIFY10B,* confirmed by PCR amplification and RT-PCR.


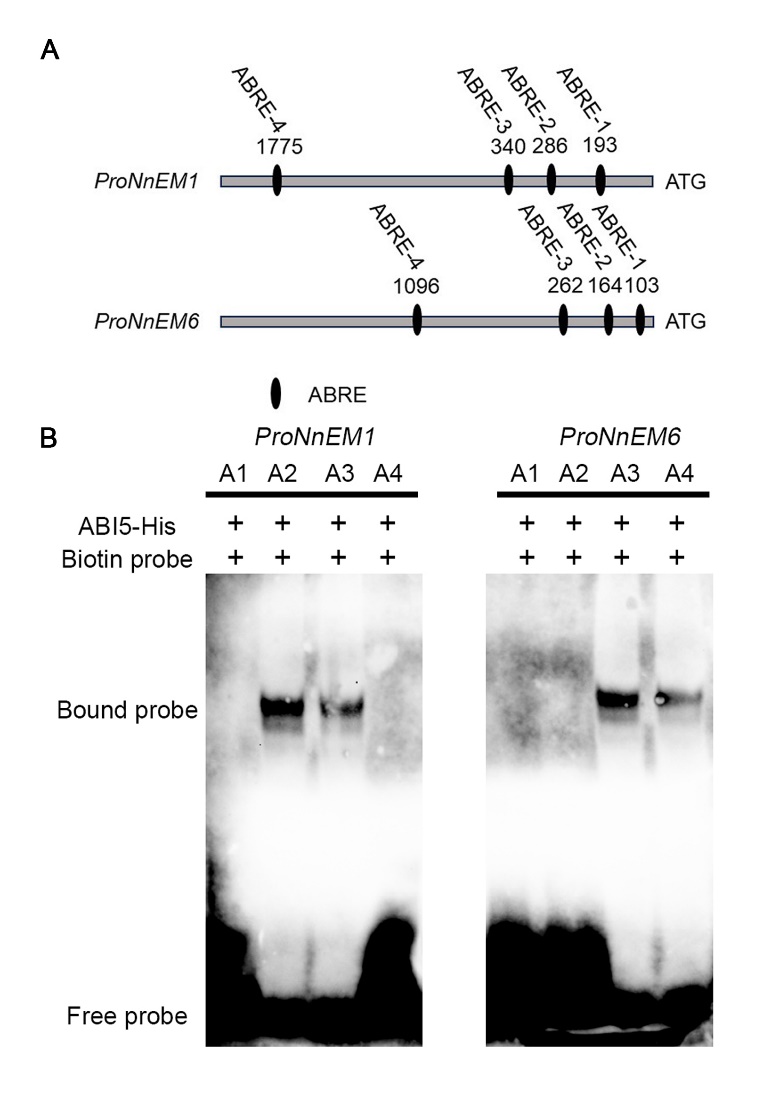


**Figure S4.** NnABI5 binds to the *NnEM1* and *NnEM6* promoters.

(A) A schematic diagram of the *NnEM1* and *NnEM6* promoters, indicating potential ABRE motifs (*NnEM1*-ABRE1/2/3/4 and *NnEM6*-ABRE1/2/3/4).

(B) EMSA results confirmed that NnABI5 binds to *NnEM1* (A2 and A3) and *NnEM6* (A3 and A4) motifs in *vitro*.


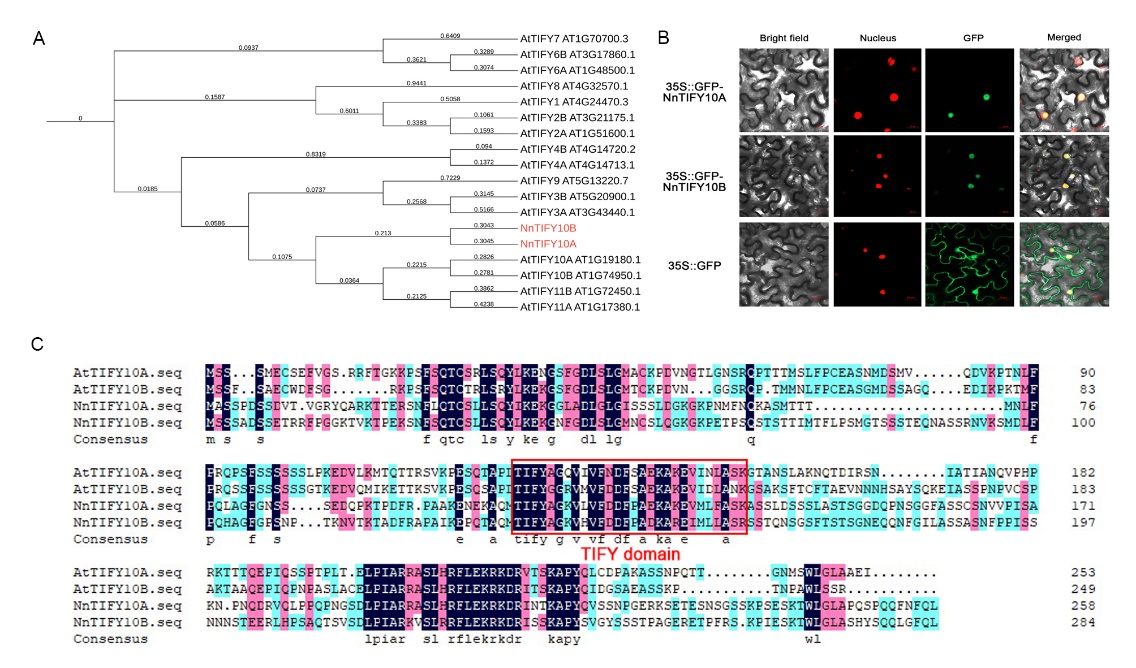


**Figure S5.** Bioinformatics analysis of NnTIFY10A/B.

(A) A Phylogenetic tree analysis of NnTIFY10A/B and related TIFY proteins in Arabidopsis.

(B) Subcellular localization of NnTIFY10A/B in *N. benthamiana* leaves based on NnTIFY10A/B-GFP fusion. Scale bar, 20 µm.


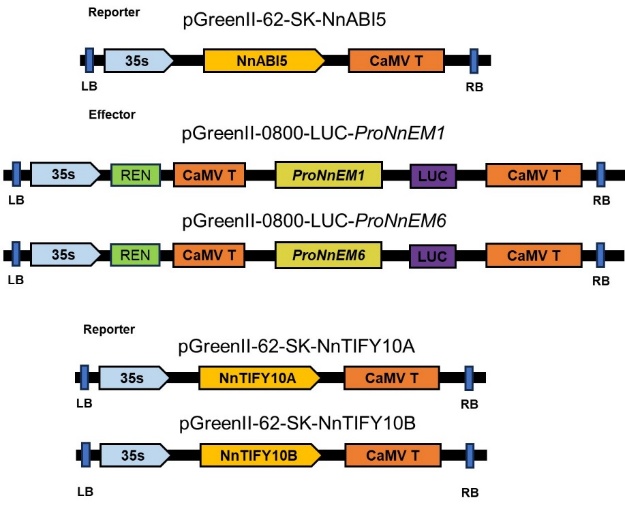
(C) An Amino acid sequence alignment of NnTIFY10A/B and AtTIFY10A/B, and the red box represents the TIFY domain.

**Figure S6.** Diagram of the pGreenII 62-SK and pGreenII 0800-LUC vectors.
