# Supplementary material for: Molecular mechanism of abscisic acid regulating bud-break through the NnTIFY10A/B-NnABI5 module in lotus
Source: Hortic Res. 2026 Apr 10;13(8):uhag125. doi: 10.1093/hr/uhag125 (PMC13404126; doi:10.1093/hr/uhag125)
Supplement: Web_Material_uhag125 [file web_material_uhag125.zip › Supplementary table.docx]

**Supplementary Table**

**Table S1.** Primers used for qRT-PCR analysis and cloning in this study.

Primer name sequence (5’ to 3’)

**RT-qPCR assays**

NnABI5-F: CAACGGCGAATCAGTATG

NnABI5-R: CTCTCCACTACCTTCTCTAC

NnTIFY10A-F: AGAAAGCAAAGGAGGTCAT

NnTIFY10A-R: CTCGGTCTTGGTTAGGATT

NnTIFY10B-F: CCTATCTCAAGCAACAACAA

NnTIFY10B-R: TGGAACTGGAATAGCCTAC

NnEM1-F: CTAGGCACGGAAGGATAT

NnEM1-R: CGGTTCTTAGTGAACTTGG

NnEM6 -F: GGAAGGATACCAGGAGATG

NnEM6-R: TGGATTCGTCGATGTCAA

NnABI5Like-F: CAACGGCGAATCAGTATG

NnABI5Like-R: CTCTCCACTACCTTCTCTAC

NnABF2-F: ATACGGTTCATTCTCAGTCT

NnABF2-R: GCAATTCCACCTCTAATAGC

NnABF4-F: CGTTCATACTCAGTCTCCTA

NnABF4-R: CTGGTTGGTGTTTGCTAAA

NnACTIN-F: GCGTTCTGCCGTCTTCTAAA

NnACTIN-R: CCCTCTTGGATTGTGCCTC

NnPYL1.1-F: GGAATACGGACGATGACA

NnPYL1.1-R: AAAGAGTAAAGCCCTGAGT

NnPYL1.2-F: TGGATATACTGGATGATGAGAG

NnPYL1.2-R: GGCACATCTACTACATACGA

NnPYL2-F: GGCGATGTAGGTACTCTC

NnPYL2-R: TAATTGGTGAGGCGATGT

NnPYL4.1-F: CTCTCCACCCTTCCTCTA

NnPYL4.1-R: AAGTTCTCGGCAATCTGA

NnPYL4.2-F: CATCGGCTCAGCAATTATC

NnPYL4.2-R: GCGAACAAGCAAGTATCC

NnPP2C75-F: TGAGAGGATGGATGAAGTG

NnPP2C75-R: GCAGTTGGCAACAATGATA

NnPP2C051-F: GTAGCGATACCACTCTCC

NnPP2C051-R: GAAGTAGCAAGCACTCCTA

NnPP2C06.1-F: CAAGAAGCGAAGATGATGAA

NnPP2C06.1-R: AGTAAGATGGTCAGCAACA

NnPP2C06.2-F: TGATGTCCAATGATGAGGT

NnPP2C06.2-R: TCCACAACAATGACTGAGA

NnPP2C2A.1-F: GGAACGAAGTGAATGAAGG

NnPP2C2A.1-R: CGATTACGACTACGCTCA

NnPP2C2A.2-F: CAAGGAGGTGCTAGTAGG

NnPP2C2A.2-R: CAGACGAGAGAGGAACAG

NnPP2C16-like-F: TTCCAGAACCAGAAGTCAT

NnPP2C16-like-R: TTGTGCCAGAGAAGGATT

NnAOS1-F: GATGAAGCGAAGAAGATGG

NnAOS1-R: TCCGTTAGAGCGTATAGC

NnACX2-F: TCTACTTCGCCAGCAATT

NnACX2-R: TGAACATCTCCTACCACATT

NnJMT1-F: AGAAGAATTGCCACCAGT

NnJMT1-R: CTCAACCTCAAGGATGTCTA

NnJMT4-F: GATTTCTCGCTGTTTCTCAA

NnJMT4-R: ATCCAACTTCTCCTCTTCAA

NnLOX2.1-F: AGAGGAATGGCAGTTGAG

NnLOX2.1-R: GGAGTTCTTGGTCGGATT

NnLOX2.2-F: TGGCTGTCCTAATCGTATT

NnLOX2.2-R: GCTCCTGTTCTGTTCTTC

NnLOX3-F: ATACGAACCGACACAGAG

NnLOX3-R: GTCAGGATGGAGACAAGAT

NnLOX6-F: CCGTGTGGCATTAGACTA

NnLOX6-R: CATCAGAGGAGACGCTAC

AtABI5-F: GAATGGTTGGACCGTTAAG

AtABI5-R: CATCCTCCTCTGTCTTCTC

AtEM1-F: GAGATGGGTCACAAAGGT

AtEM1-R: ATCGTACTGAGTCCTCCT

AtEM6-F: CGAAAGGAGCAGTTAGGA

AtEM6-R: GTGGGGAAGTTTGATTTAGG

**Genetic transformation**

NnABI5GFP(OX)-F: TTGATACATATGCCCGTCGACATGGTGGTTCCAGAGTCGGAA

NnABI5GFP(OX)-R: TCAGAATTCGGTACCCCCGGGCGAGATTTGTCCAGTGGGGC

NnABI5(pIR)-F: GCAGAATCTGAATTCGTCGACATGGTGGTTCCAGAGTCG

NnABI5(pIR)-R: GCTCGAGAAGCTTGTCGACCGAGATTTGTCCAGTGGG

NnABI5(IR-X-RI)-F: AATCTGAATTCGTCGACAAGCTTCACCGGTGTGTCCAGTTA

NnABI5(IR-X-RI)-R: TAGCCTAGGCTCGAGAAGCTTCATCGACTTAAGCTTCTCTGC

NnTIFY10AGFP(OX)-F: TTGATACATATGCCCGTCGACATGGCAAGTTCGCCGGAT

NnTIFY10AGFP(OX)-R: TTCGAATCCGGTACCCCCGGGTAGTTGGAAGTTAAATTGCTGTGGA

NnTIFY10A(pIR)-F: GCAGAATCTGAATTCGTCGACATGGCAAGTTCGCCGGAT

NnTIFY10A(pIR)-R: GCTCGAGAAGCTTGTCGACTAGTTGGAAGTTAAATTGCTGTGGA

NnTIFY10A(IR-X-RI)-F: AATCTGAATTCGTCGACAAGCTTGCAAGTAAGGCAAGCTCC

NnTIFY10A(IR-X-RI)-R: TAGCCTAGGCTCGAGAAGCTTGTTAAATTGCTGTGGAGATTGT

NnTIFY10BGFP(OX)-F: TTGATACATATGCCCGTCGACATGTCCAGCTCTGCGGATTCG

NnTIFY10BGFP(OX)-R: TTCGAATCCGGTACCCCCGGGCGATAATTGGAATCCCAATTGC

NnTIFY10B(pIR)-F: GCAGAATCTGAATTCGTCGACATGTCCAGCTCTGCGGATTCG

NnTIFY10B(pIR)-R: GCTCGAGAAGCTTGTCGACCGATAATTGGAATCCCAATTGC

NnTIFY10B(IR-X-RI)-F: AATCTGAATTCGTCGACAAGCTTGCTTCTAGCTAGTAGAAGCAGTACC

NnTIFY10B(IR-X-RI)-R: TAGCCTAGGCTCGAGAAGCTTGCGAATAATGTGAAGCCAAG

**Y2H assays**

NnABI5(PGAD)-F: GTACCAGATTACGCTCATATGATGGTGGTTCCAGAGTCGGAA

NnABI5(PGAD)-R: CAGCTCGAGCTCGATGGATCCCGAGATTTGTCCAGTGGGGC

NnTIFY10A(PGAD)-F: GTACCAGATTACGCTCATATGATGGCAAGTTCGCCGGAT

NnTIFY10A(PGAD)-R: CAGCTCGAGCTCGATGGATCCTAGTTGGAAGTTAAATTGCTGTGGA

NnTIFY10A(PGAD)-N-R: CAGCTCGAGCTCGATGGATCCGGAGCTTGCCTTACTTGC

NnTIFY10A(PGAD)-C-F: GTACCAGATTACGCTCATATGATGCTCGATTCCAGCAGCTT

NnTIFY10B(PGAD)-F: GTACCAGATTACGCTCATATGATGTCCAGCTCTGCGGATTCG

NnTIFY10B(PGAD)-R: CAGCTCGAGCTCGATGGATCCCGATAATTGGAATCCCAATTGC

NnTIFY10B(PGAD)-N-R: CAGCTCGAGCTCGATGGATCCGGTACTGCTTCTACTAGCTAGAAGC

NnTIFY10B(PGAD)-C-F: GTACCAGATTACGCTCATATGATGCAGAATTCCGGTAGCTTC

NnABI5(PGBD)-F: TCAGAGGAGGACCTGCATATGATGGTGGTTCCAGAGTCGGAA

NnABI5(PGBD)-R: CCGCTGCAGGTCGACGGATCCCGAGATTTGTCCAGTGGGGC

NnABI5(PGBD)-N-R: CCGCTGCAGGTCGACGGATCCTGGCTCTCCCAAAACCGTAC

NnABI5(PGBD)-C-F: TCAGAGGAGGACCTGCATATGATGTCGAATTATGCACCAAATGG

NnTIFY10A(PGBD)-F: TCAGAGGAGGACCTGCATATGATGGCAAGTTCGCCGGAT

NnTIFY10A(PGBD)-R: CCGCTGCAGGTCGACGGATCCTAGTTGGAAGTTAAATTGCTGTGGA

NnTIFY10B(PGBD)-F: TCAGAGGAGGACCTGCATATGATGTCCAGCTCTGCGGATTCG

NnTIFY10B(PGBD)-R: CCGCTGCAGGTCGACGGATCCCGATAATTGGAATCCCAATTGC

**BIFC assays**

NnABI5(YFP^C^)-F: TGGCGCGCCACTAGTGGATCCATGGTGGTTCCAGAGTCGGAA

NnABI5(YFP^C^)-R: AGCGGTACCCTCGAGGTCGACCGAGATTTGTCCAGTGGGGC

NnTIFY10A(YFP^N^)-F: TGGCGCGCCACTAGTGGATCCATGGCAAGTTCGCCGGAT

NnTIFY10A(YFP^N^)-R: AGCGGTACCCTCGAGGTCGACTAGTTGGAAGTTAAATTGCTGTGGA

NnTIFY10B(YFP^N^)-F: TGGCGCGCCACTAGTGGATCCATGTCCAGCTCTGCGGATTCG

NnTIFY10B(YFP^N^)-R: AGCGGTACCCTCGAGGTCGACCGATAATTGGAATCCCAATTGC

**Pull-down assays**

NnABI5(PGEX)-F: GATCTGGTTCCGCGTGGATCCATGGTGGTTCCAGAGTCGGAA

NnABI5(PGEX)-R: CTCGAGTCGACCCGGGAATTCCGAGATTTGTCCAGTGGGGC

NnABI5(PET32a)-F: GCCATGGCTGATATCGGATCCATGGTGGTTCCAGAGTCGGAA

NnABI5(PET32a)-R: TTGTCGACGGAGCTCGAATTCCGAGATTTGTCCAGTGGGGC

NnTIFY10A(PET32a)-F: GCCATGGCTGATATCGGATCCATGGCAAGTTCGCCGGAT

NnTIFY10A(PET32a)-R: TTGTCGACGGAGCTCGAATTCTAGTTGGAAGTTAAATTGCTGTGGA

NnTIFY10B(PET32a)-F: GCCATGGCTGATATCGGATCCATGTCCAGCTCTGCGGATTCG

NnTIFY10B(PET32a)-R: TTGTCGACGGAGCTCGAATTCCGATAATTGGAATCCCAATTGC

**Y1H assays**

PNnEM1(pHIS2)-F: GACTCACTATAGGGCGAATTCAATCATTTGATTTCACCAACATTGTA

PNnEM1(pHIS2)-R: GATTCGCGAACGCGTGAGCTCTTTTCGAACTGATTTCTTCACCAC

PNnEM6(pHIS2)-F: GACTCACTATAGGGCGAATTCCTTAAAAATTGAATTTAATTTTAAAAATTACAG

PNnEM6(pHIS2)-R: GATTCGCGAACGCGTGAGCTCTGCGTTGCTGTAGGAAAGCG

**Dual-LUC assays**

NnABI5(62SK)-F: CGCTCTAGAACTAGTGGATCCATGGTGGTTCCAGAGTCGGAA

NnABI5(62SK)-R: GTCGACGGTATCGATAAGCTTCGAGATTTGTCCAGTGGGGC

NnTIFY10A(62SK)-F: CGCTCTAGAACTAGTGGATCCATGGCAAGTTCGCCGGAT

NnTIFY10A(62SK)-R: GTCGACGGTATCGATAAGCTTTAGTTGGAAGTTAAATTGCTGTGGA

NnTIFY10B(62SK)-F: CGCTCTAGAACTAGTGGATCCATGTCCAGCTCTGCGGATTCG

NnTIFY10B(62SK)-R: GTCGACGGTATCGATAAGCTTCGATAATTGGAATCCCAATTGC

PNnEM1-LUC-F: GGGCCCCCCCTCGAGGTCGACAATCATTTGATTTCACCAACATTGTA

PNnEM1-LUC-R: CGCTCTAGAACTAGTGGATCCTTTTCGAACTGATTTCTTCACCAC

PNnEM6-LUC-F: GGGCCCCCCCTCGAGGTCGACAGACTAGGAAATATGGGAAATTTCTCA

PNnEM6-LUC-R: CGCTCTAGAACTAGTGGATCCTGCGTTGCTGTAGGAAAGCG

**Table S2.** Primers used to synthesize the EMSA probes.

PEM1-ABRE-BJ-1-F：GCCGCGTTTATCATTACGTGTCTGTTTCAGAGTTC

PEM1-ABRE-BJ-1-R：TAACAAGATAATAATGGTTATATCATATTTATATC

PEM1-ABRE-BJ-2-F：ACACGCAAAACGAAGACGTGTGTCATTATCTTTCG

PEM1-ABRE-BJ-2-R：CGAAAGATAATGACACACGTCTTCGTTTTGCGTGT

PEM1-ABRE-2-F：ACACGCAAAACGAAGACGTGTGTCATTATCTTTCG

PEM1-ABRE-2-R：CGAAAGATAATGACACACGTCTTCGTTTTGCGTGT

PEM1-ABRE-TB-2-F：ACACGCAAAACGAAGTAATGTGTCATTATCTTTCG

PEM1-ABRE-TB-2-R：CGAAAGATAATGACACATTACTTCGTTTTGCGTGT

PEM1-ABRE-BJ-3-F：CCATAAGCTGAAAGGTACGTGTAACTCAGAAACAA

PEM1-ABRE-BJ-3-R：TTGTTTCTGAGTTACACGTACCTTTCAGCTTATGG

PEM1-ABRE-3-F：CCATAAGCTGAAAGGTACGTGTAACTCAGAAACAA

PEM1-ABRE-3-R：TTGTTTCTGAGTTACACGTACCTTTCAGCTTATGG

PEM1-ABRE-TB-3-F：CCATAAGCTGAAAGGTTAATGTAACTCAGAAACAA

PEM1-ABRE-TB-3-R：TTGTTTCTGAGTTACATTAACCTTTCAGCTTATGG

PEM1-ABRE-BJ-4-F：TTTAAACCTAATATGCACGTGTGAGATGAAAAGATT

PEM1-ABRE-BJ-4-R：AATCTTTTCATCTCACACGTGCATATTAGGTTTAAA

EM6-ABRE-BJ-1-F：CTATAAACAACTGAAAACGTGTCGTTCATCTCAGCTT

EM6-ABRE-BJ-1-R：AAGCTGAGATGAACGACACGTTTTCAGTTGTTTATAG

EM6-ABRE-BJ-2-F：GGACACGCAGTGGCAGACGTGGCGAAGGAAGAATGA

EM6-ABRE-BJ-2-R：TCATTCTTCCTTCGCCACGTCTGCCACTGCGTGTCC

EM6-ABRE-BJ-3-F：TGCTGTTTTCTGAGCAACGTGACTGTGCTGCATCCT

EM6-ABRE-BJ-3-R：AGGATGCAGCACAGTCACGTTGCTCAGAAAACAGCA

EM6-ABRE-3-F：TGCTGTTTTCTGAGCAACGTGACTGTGCTGCATCCT

EM6-ABRE-3-R：AGGATGCAGCACAGTCACGTTGCTCAGAAAACAGCA

EM6-ABRE-TB-3-F：TGCTGTTTTCTGAGCATAATGACTGTGCTGCATCCT

EM6-ABRE-TB-3-R：AGGATGCAGCACAGTCATTATGCTCAGAAAACAGCA

EM6-ABRE-BJ-4-F：ATATGAAAAGCTATAACGTGTGTAGGATTGGCAAC

EM6-ABRE-BJ-4-R：GTTGCCAATCCTACACACGTTATAGCTTTTCATAT

EM6-ABRE-4-F：ATATGAAAAGCTATAACGTGTGTAGGATTGGCAAC

EM6-ABRE-4-R：GTTGCCAATCCTACACACGTTATAGCTTTTCATAT

EM6-ABRE-TB-4-F：ATATGAAAAGCTATATAATGTGTAGGATTGGCAAC

EM6-ABRE-TB-4-R：GTTGCCAATCCTACACATTATATAGCTTTTCATAT

**Table S3.** Bud length data corresponding to Figure 2E, 3D and 6F.

|  | 0 d | 7 d | 14 d |
| --- | --- | --- | --- |
| WT | 2.15 | 3.52 | 4.87 |
|  | 2.09 | 3.78 | 4.66 |
|  | 2.03 | 3.61 | 4.53 |
| EV | 1.97 | 3.67 | 4.59 |
|  | 1.92 | 3.74 | 4.69 |
|  | 2.08 | 3.81 | 4.73 |
| NnABI5-pIR | 2.07 | 3.12 | 3.74 |
|  | 1.85 | 2.81 | 3.54 |
|  | 1.98 | 3.05 | 3.62 |
| IR-NnABI5-IR | 2.16 | 4.32 | 5.89 |
|  | 1.93 | 4.21 | 5.23 |
|  | 1.99 | 4.25 | 5.66 |

|  | 0 d | 7 d | 14 d |
| --- | --- | --- | --- |
| WT | 1.89 | 3.48 | 4.59 |
|  | 2.18 | 3.83 | 4.73 |
|  | 1.97 | 3.58 | 4.66 |
| EV | 2.06 | 3.52 | 4.72 |
|  | 1.94 | 3.65 | 4.56 |
|  | 2.22 | 3.79 | 4.75 |
| NnEM1-pIR | 1.82 | 2.87 | 3.34 |
|  | 2.13 | 3.08 | 3.43 |
|  | 1.99 | 2.99 | 3.41 |
| IR-NnEM1-IR | 1.76 | 2.94 | 5.17 |
|  | 2.09 | 2.83 | 5.35 |
|  | 2.24 | 3.11 | 5.57 |
| NnEM6-pIR | 1.91 | 4.18 | 3.23 |
|  | 2.04 | 4.36 | 3.46 |
|  | 1.85 | 4.23 | 3.23 |
| IR-NnEM6-IR | 2.17 | 4.29 | 5.98 |
|  | 1.97 | 4.15 | 5.64 |
|  | 2.11 | 4.38 | 5.85 |

|  | 0 d | 7 d | 14 d |  |
| --- | --- | --- | --- | --- |
| WT | 1.79 | 3.68 | 4.23 |  |
|  | 2.21 | 3.52 | 4.55 |  |
|  | 1.98 | 3.65 | 4.42 |  |
| EV | 2.14 | 3.79 | 4.55 |  |
|  | 1.84 | 3.43 | 4.27 |  |
|  | 2.07 | 3.76 | 4.39 |  |
| NnTIFY10A-pIR | 1.92 | 4.28 | 5.26 |  |
|  | 1.78 | 4.24 | 5.14 |  |
|  | 2.19 | 4.31 | 5.52 |  |
| IR-TIFY10A-IR | 2.03 | 3.14 | 4.03 |  |
|  | 1.96 | 2.85 | 4.01 |  |
|  | 2.23 | 3.02 | 4.11 |  |
| NnTIFY10B-pIR | 1.87 | 4.19 | 5.52 |  |
|  | 1.81 | 4.33 | 5.31 |  |
|  | 2.11 | 4.26 | 5.63 |  |
| IR-TIFY10B-IR | 2.16 | 2.97 | 4.05 |  |
|  | 1.99 | 3.09 | 3.92 |  |
|  | 2.08 | 2.89 | 4.12 |  |

**Table S4.** Gene id and full-length sequences of 16 candidate genes

ncbi_104588466 (ABI5)

ATGGTGGTTCCAGAGTCGGAAGCTCGCGGTCGTGTAGAGGTGGAAATAACGACAGCAAAGCCACCCGAGGAGGTACCAAAGACGCACTCGTTGTCATCGCTTGGAAGACAGTCTTCCATCTACTCTCTTACTCTGGACGAGTTCCAGCACACATTATGCGAGAACGGGAAGAGCTTCGGATCAATGAACATGGACGAATTCCTCAACAACATCTGGACTGCGGAAGAGAACCAGGCTATTGGCTGCAACAATGAGAGGTTGCAGTCCAACCCAGACGAAGCCGCAGCTAACAGGGCCAACTCCGGACAACCCAGCGTACTCCGACAAGGTTCAATCACCCTCCCTGCTCCACTATGCCACAAAACTGTCGACGAAGTCTGGGCCGAAATCCACAAACACCAAGAGCGGCAACAACAGGAGCAGCAACCAAACAACGGAGGTAATCCCGGAACTACACAGCGCCAACTCACATTCGGAGAGATGACTCTCGAGGATTTCTTAATAAGAGCTGGGATTGTTCGGGAGGCTTGTGGGCCCTCATCTAAGCACCAGCTTCAGACTCCGGCGACTGTTCCACCTACTGGGACGGCGACACAGCAGTATGGGTTATACCAGAATAATAACGCGGGCGTGGACCCCAACTTCGGAATGGGACCCTTATTGGGTCTTGGATTCTCCGAGCAACGGAGTACTGGAAACGGCGTGGCAAACGGTGTCCTGTCGTACCAAACATTCCCGCAAAGCGGTACGGTTTTGGGAGAGCCATCGAATTATGCACCAAATGGAAAGAGGAACGGTGGTTTTCCAGCGACGGTTTGTTTTGGCGGTAGAACAGGAAATGGAGCTGGATATGTAGCAGCAGCAGCGGCAGCGAATACGTTGGGGCCACCGGTGTGTCCAGTTACGTCTGAAGGGATGTGCACGAGTCAAGTGGATAACGCAACGGCGAATCAGTATGGAGTGGATATAAGTGGGATAAGGGGGAGAAAGAGGTTAATAGACAGTCCAGTAGAGAAGGTAGTGGAGAGGAGGCAACGCAGGATGATCAAGAACAGGGAGTCGGCGGCCAGGTCTCGAGCCAGGAAACAGGCCTATACGGTGGAGCTGGAAGCAGAATTGAATCATCTCAAAGAGGAGAATGGTCGCCTCAAAGAAGCTTTGGCAGCTTCTGAGAAGAAAAGGAGAGAGGAGCTGGAAGAAAAGATGAAGACGAAGCCTCCAACAAAGGCGCAGAAAACTGCAGAGAAGCTTAAGTCGATGAAGAGGACCGTGAGCTGCCCCACTGGACAAATCTCGTAA

ncbi_104588466 (ABI5-Like)

ATGGTGGTTCCGGAGTCGGAAGCTCATGCCCGTGGAGAGGTAGAAGCAACAGCAGAACAGCCACCCGAGGATGCATCGAAGACCCACTCCCTGTCATCACTTGGAAGACAATCCTCCATCTACTCCCTCACCCTCGACGAGTTCCAGCACGCACTGTGCGAGAACGGGAAGAATTTTGGGTCCATGAACATGGACGAGTTCCTCAATAACATCTGGACCGCCGAAGAAAACCAAGCTATTCACTCCAACAATGAGAATTCGCAAACCAACCCAGACGAAGTCAGAGCCGGTAAGCTAAGGTGCAACCAACCCGGATTGCTCCGCCAAGGTTCGCTCACTCTCCCTGCACAGCTCTGCCACAAAACCGTCGACGAGGTATGGGCTGAAATCCACAAAATCCAAGAACAGCAACAGCTAGAGGGTCAACAACTGCAGGACTCTGGTGATCCTGGAACTACCCAGCGTCAAATCACTTTTGGAGAGATGACTTTGGAGGATTTCTTAATCAGAGCTGGGGTTGTCCGGGAAACATGTGGATCCTCCTCTCAGCAACACCGACTGATGACGCCAGCTACTGGTCTACCACCTGCAACGGCGACACCGCAGTATGGGTACTCCGACCACCAGAGCACCGGAAACGGCGTAGAAAGCGGTGTCCCAACGTACCAAACATTCCCACAAAGTGGTTCAGGAGAACCATCAAACTATACGGCGAATGGAAAGAGGAACAGCGATTACCAGCAAACAGGAGTTTGTTTTGGAGGAAGAGAGGGAAATGGAGCTGGATATGCAACAGCTGTGACAAATACGCTGGGGTCGCAGGTGAACTCGGCGACGTCTGATGGGATATGCACGAATCAAGTGGACAACACGGCAGCGACTCAGTACGGGATGGATATGGGTGGAGATGGAATAAGGGGGAGGAAGAGGATACTGGACGGTCCAATAGAGAAAGTGGTGGAGAGGAGGCAACGCAGGATGATCAAGAACAGAGAATCAGCGGCCAGGTCTCGAGCCAGAAAACAGGCATATACTGTGGAGCTGGAAGCAGAGTTGAACCATCTGAAAGAGGAGAATGCTCGGCTCAAGATGACTCTGGCAGAAGCAGAGAAGAAAAGAAGGCAAGAGATGGAGGAAGAAATGAAGGTGAAGCCTCCAACAAAGGCGCAGAAAGCTGCCGATAAGCTGAAGGCAATAAGGAGGACTGTGAGCTGCTCCAATGGGTTCTCTTAA

ncbi_104601772(ABF2)

ATGAACTTCAAGAACGCTGGTGATGTACCACCACCGGAAGGCAGCGGCGGCAGTGGAAGGCTGTCGGGAAATTTCCTGTTGGCCAGACAGCCTTCCATCTACTCGTTAACCTTCGATGAGTTCCAGAACACCATGGGTGGCCTTGGCAAGGATTTTGGCTCCATGAACATGGACGAATTTCTCAAGAATATATGGACCGCTGAAGAGGCTCAAGCCATGGCCTCCTTTGGAGGGGCCGAAGGTGGGTTCCCTGGCGGGGGCCTGCAGAGGCAGGGTTCCTTAACTTTGCCTCGGACTCTCAGCCAGAAGACAGTTGATGAAGTCTGGAGAGACATATTTAAAGAAAGTGGTGGTGGCAAGGACGGAAATAGCAATGGGGGATCAAATTTGCAGCAGAGGCAGCAAACTTTAGGGGAGATGACTCTGGAAGAGTTCTTGGTCAAAGCAGGGGTAGTAAGAGAAGATACTCAAGCAGCTGGACGACTGAATAATAGCGGATTCTGTGGGGAGATATCATGTCCCAATGACATTATCAGTCTAGCTCTTGGGTTTCCACAGCCGGGACGGACTAACGGAATCGTGTCTAATTGCGCCACTGAGAACATCAATACGGTTCATTCTCAGTCTCCTAGTCTAGCCATCAATACAAACGGGGTCGGATCTTGTCAGCCGTCGCTGCAGCAGCAGCAACAGTACCTACAACGAATATTCCCAAAGCAACCCACGGTGGCTTATGCCTCCCCAATGCATTTAGGAAACACTAACCAGCTGTCTAGCTCTGCTATTAGAGGTGGAATTGCAGATTCAACGATGAATAACTCTTTTGGTCAGGGTGGAGGAATGGGCACTGTTGGTATAGGGGCTGGGGCTGTTACAGTTGCAACAGGATCTCCAGCAAACCAGTTATCCTCTGATGGACTTGGGAAGAGCAATGGCGATACACCTTCCTTGTCGCCAGTACCTTATCCATTTAATGGAGGTCTTAGAGGAAGAAGATGCAGCGGAGCTGTGGAGAAGGTGGTGGAGCGGAGGCAGAGAAGAATGATTAAGAACAGAGAGTCAGCTGCAAGATCACGCGCTCGAAAGCAGGCATATACCATGGAATTGGAAGCAGAAGTTGCAAAACTTAAAGATGAGAACCAAGAATTGCAGAAGAAACAGGCGGAAATTATGCAAATGCAGAAAAATCAGGTTTTGGAGATGATAAATCAGCAATGGGGAAATAATAAAAAGCAATGCTTAAGGAGGACACAAACTGGTCCATGGTAG

ncbi_104606250(ABF4)

ATGGGATCTCACATGAACTTCAAGAACGTTGGTGATGCACAACCACTGGAAGGCAACACAGGCAGTGGAAGGCCGCCGGGAAACCTCCCGTTGGCCCGGCAGCCTTCTATCTTCTCGTTAACCTTAGATGAGGTTCAGAACACCATGGGTGGCCTTGGAAAGGATTTTGGCTCCATGAACATGGATGAATTTCTCAAGAATATTTGGACTGCCGAAGAGGCTCAAGCCATGGCATCCACCTTCGGAGGGACTGAAGGAGGGGTCCCTGGCACGAATCTGCAGCGGCAGGGTTCCTTAACTTTGCCACGGACTCTCAGTCAAAAGACGGTCGATGAAGTATGGAGAGACATATTTAAAGAAAGTGCTAGCGGCAAAGATGGGAGTGGTAGTGGGGGATCGAATTTGCAGTCGAGGCAGCAAACTTTAGGGGAGATGACTCTGGAGGAGTTCTTGGTGAAAGCAGGAGTGGTGAGAGAAGATATTCAAACAGGAACACCGAATAATAGCGGGATGTATGTTGAGACGCGTTCCAATGACAATATTGGTCTGGCTCTTGGGTTTCCACAGCCAGGACAGACCAACGGAGTTGTGCTTAACCGAATTGCAGAGAGTAACAATCCCGTTCATACTCAGTCTCCTAATCTAGCCATAAATATAAACGGAGCCAGATCTTCTCAGCCGCAACAGCAGCAGCAGCTACCCCAACTCTTCGCAAAACAATCTACGGTGGCTTTTGCCTCCGCAATGCATTTAGCAAACACCAACCAGCTGTCTAACCCAGGAATGAGGGGTGGAGTTGCAGATCCAATGTTGAACAATGGTTTTGGTCAAGGCGGTGGACCACAGAGTGGAGGAATGGGCATGGTTGGTCTAGGGGCCGGGGCTGTTACAGTTGCAACGGGATCTCCAGCAAACCAGTTATCCTCTGATGGGCTTGGGAAGAGCAATGGTGATACATCTTCCTTGTCACCAGTACCTTATCCTTTTAATAGTCTAAGAGGGAGAAGATGCAGTGGGGCTGTGGAGAAGGTAGTGGAGCGGAGGCAGCGAAGAATGATCAAGAATAGAGAGTCAGCTGCAAGATCACGGGCTCGAAAGCAGACTTCTTAG

ncbi_104597274(PYL4.1)

ATGCCGGCAACACCTCCCAAATCATCTCTCCAAGTCCACAGAATCAATAACACAATCACCTCCGCTACCAACACAACCAACGTTCTCTCCCAGAAACAATCACAAGCCACCACACTCTCCCGCCGGGTCCCTCTATCCTGCGCCATTCCGGTCCCCGACTACATCGCGCGCTACCACACCCACGCCGTTGGGCCCAACCAGTGCTGCTCCTCCGTCATCCAACCCATCGCCGCTCCCGTCTCCACCGTATGGTCCGTCGTCCGCCGCTTTGACAATCCTCAAGCCTACAAGCACTTCGTCAAGAGCTGCCACGTTCTCGTCGGAGACGGTGATGTCGGTACTCTCCGAGAGGTCCATGTCGTTTCTGGTCTCCCCGCCGTGAAAAGCACCGAGCGCCTTGAGATCCTCGACGACGAGCGCCACGTCCTTAGCTTCAGCGTCATCGGTGGGGAACATCGCCTCTCCAATTACAGATCAGTCACCACTCTCCACCCTTCCTCTACTGGCGCTGGAACCGTTGTTGTCGAATCGTACGTCGTTGACATCCCACCAGGGAATACCAGCGAAGACACATGTATGTTCGTCGACACCATAGTCCGCTGCAACTTACAATCACTCGCTCAGATTGCCGAGAACTTAGCCGGCCGGAACCGGACATCGTCGTGA

ncbi_104602513(PYL2)

ATGCCTGCAACACCTCCAAAATCATCTCTCCAAACCCACAGAATCAATAACACAATCACCAACATGATCTGCCACAAACAATCACAACCCACTCCACTTTCCCGCAGGTTCCCCCTATCTTGCGCCACTTCCGTGCCCGACTACATCGTGCGCTACCACACCCACGCCGTAGGACCCAACCAGTGCTGTTCTTCCGTCATCCAACCAATTGCCGCTCCTGTCTCCACTGTCTGGTCCGTCGTCCGCCGCTTCGACAACCCTCAAGCATACAAGCACTTCGTCAAGAGCTGCCACGTTATCGTTGGCGACGGCGATGTAGGTACTCTCCGAGAGGTCCACATCGTTTCTGGTCTCCCCGCCGAAACAAGTACCGAGCGCCTTGAAATCCTCGACGACGAACGCCACGTCCTCAGCTTCAGCGTTATCGGTGGGGAACATCGCCTCACCAATTACAGATCCATCACCACTCTCCACCCGTCGTCTTCTGGCACCGGAACCGTCGTCGTCGAATCGTACGTCGTTGACATCCCGCCAGGCAATACCGGTGAAGATACTTGTATGTTCGTCGATACCATAGTCCGTTGCAACTTACAATCGCTTGCTCAGATCGCCGAGAGCTTGACCTCCCGGAACCGTACATCGTCATGA

ncbi_104608779(PYL1.1)

ATGATAGTAATGGAGGAAGAAGAGATATCAGCGACGGAGAAGGAGAAGATGGAATACACGAACACAGCACATCACACAAGGATTCCAGCCGGTCTAAGTGGAGAAGAATTCGAGGAATTAAAGCCAGTCATCTACCACCACCATACGTATCGTATCTGTACGGGTAAATGTTCATCTCTACTCGCTCAACGGATACAAGCTCCCATCGACACGGTCTGGTCCATAGTTCGTCGATTCGATAAGCCGCAGACCTACAAGCATTTCATCAAGAGCTGCTTCCTTAAAGAAGGATCCCAGCAGTACCCCACCACCGTCGGATGCTTGAGAGAGGTAAACGTTATATCCGGGCTTCCGGCGGCGACCAGTACGGAGAGGTTGGATATTCTGGACGACGAGAGACACGTCACTGGGTTCAGCATCATCGGCGGGGAGCACCGGTTGAAGAACTACCGGTCGGTGACGACGGTGAATGAGTGTAAAGGGGGCGACGGGAGGATCCGGACCGTTGTTTTGGAGTCGTACGTTGTGGATGTGCCGGAGGGGAATACGGACGATGACACTCGTATGTTTGCCGATACGGTGGTGAAATTGAATCTGCAGAAGCTGGCGTCGGTGACCGAAGGGTTAGCTCGTGATGCGAATAGAAGAAACTCAGGGCTTTACTCTTTTTGCGTGGAATAG

ncbi_104610351(PYL1.2)

ATGGAGGAAGAAGGAGAAGTATGGACGACGGAAGAGGAAGAACAAACATACACAGCTCACCATACAACGATGCCAGCCGGCTTGATAGAAGAAGAATTCGAAGAATTGAAGCCGATCATCAACGACTTCCACACGTATCGTATCGGCTCGAGCAAATGCTCATCTCTACTCGCCCAACGTATACAAGCTCCGCTGGACACAGTCTGGTCCGTCGTCCGTCGATTCGATAAACCGCAGACGTACAAGCATTTCATCAAGAGCTGCTCGCTTAAAGAAGGGTCCCAGTCTCCCACCACCGTCGGATGCCTGAGAGAGGTCAACGTTATATCTGGGTTACCGGCGGCGACCAGTACGGAGAGATTGGATATACTGGATGATGAGAGACACGTGACAGGTTTCAGCATCATCGGCGGAGAGCACCGGTTGAGGAACTACCGATCGGTGACGACGCTGAACGAGTTCAGAGGAAGTGATGGGAGGATCTGGACTGTCGTTTTGGAGTCGTATGTAGTAGATGTGCCGGAGGGGAATACCGACGATGATACTCGCATGTTTGCTGATACGGTGGTGAAATTAAATCTGCAGAAGCTGGCGTCGATGACAGAAGGTTTAGCTTGTGACAACGACAGAAGAAACACAGGTGCGTGA

ncbi_104611581(PYL4.2)

ATGCTCTACACCCAAAATCCGAACCAGAGACAAACATACAAGGATCCCAATATGGTGGAGATGGTAACTCGGTACCACATCCATGATCTCTCGCCCAACCAGTGTAGCTCCAGCCTCGTTCAAACCATCGACGCGCCTCTGCCTCTTGTGTGGTCCGTGGTTCGTCGCTTCGACAACCCACAAGCCTACAAGCGGTTCGTGAGGAGTTGCTCTTTGCTGTATGGCGATGGTGGCATCGGTAGCCTGCGGGAGGTGCAGGTGGTGTCGGGCCTCCCGGCAGGGACCAGCATGGAGCGGCTGGATGCGCTGGATGATGAGTCGCATGTCATGGGTTTTAGCATTGTGGGTGGCGACCATCGGCTCAGCAATTATCGGTCGATCATGAGTCTTCACGATAACGGAGAAGGTGGTGGTGGGAAGACGGTGGTGGTGGAGTCTTATGTGGTTGATGTGCCGCCGGGGAGTACCAAGGAGGATACTTGCTTGTTCGCCGATACGATTGTAAAGTGCAACCACAGGTCGTTGGCTAGGATTGCAGAGAAAATGGCTTCACCATAG

ncbi_104590808(PP2C75)

ATGTCAATGGCTGAGGTTTGTCGAAAAATGGTGAGTAGCGAAGGCTCCTCCGCAAACTGCCGGGAAGCTCGCCGGCGGCGAATGGAGCTAAGGAAACTTGCTGGTGTCGCCGTAGTGCCCGGTTCGGAGTCACCTTCACGGGACAGTTTAAGTTGTAAGGAGAAACGCCAAGATAATTCCAATCATTCGGATAAGAGGAAAAGAAATCGAACGAGTGAAATTTCTGACTGTCCATCTCTATCCTCTTCATCACCGTCGTCAGAAAGCCGGGAAGCAGGAACTTCGTCGACTATGCGGTCGACTTCAGTCCAGATTTTACTTGACGATACAGTTCCGGCTTTTGGTTCGATATCGGTCTCCGGGCGTTCGCGTGAGATGGAGGATTGCTTATATGTACGAGTAAACCTCTGCCGGCCTGAGATTACCTGCGGTCGACCGCTGCATTTCTTTGCTGTGTATGACGGCCATGGTGGTTCTCACGTGGCTGAAATGTGTAAGGAGCGGATGCACGTGTTCTTAGAGGAGGAGTTGATGCGGGAGGAGTCGGAATCCGGTGAGGATGGTAGAACCGCGCAACAGGAGCGGTGGAGGTCGGCGATGGGGAGGTGCTTTGAGAGGATGGATGAAGTGGCGCTGAACGCGTGCGCATGCGGGAAAGTGGTGACGCCTTGCGAGTGTGAACGAGCTGGGTTGACGTCGGAGATAGTCGGGTCGACGGCTCTGGTGGGTGTTATCACACCGGATAGTATCATTGTTGCCAACTGCGGGGACTCGCGCGCAGTTCTCAGCCGTGGAGGGAAAGCCATACCTCTATCTTTCGATCACAAGCCGGACAGGCCGGATGAGCTGTCTCGAATCGAAGCGGCCGGGGGACGGGTAATCTACCTGAACGGAGCTCGAGTCCTCGGAATTCTGGCAATGTCCCGGGCTCTAGGTTGTTTTGGTCCCACAAGATTGGATCCGACGGTGTGCTGTCGCATTCCTATTTGTGCAATTGCAACATCTAGGAAAAGAACCTCAGAAGGAATCACGAGTGAATGA

ncbi_104592057(PP2C06.1)

ATGGATCCGTTGGACGACGAAGATCTCCATCAAGTCGGATCTCACCGGGAATCCGACGTGGCATCGAATGATTCCGGCGTATCTTCACTCTTGGGGACAGAAGACAGTAGAAGCACCACTAGCTCCAGCGACATCTCCGGCGGTAGTGGCAGCTCCGGCGAGATTCCGACCGCAGTCGTCGCAGAGGCAGTAGTGCCGAGGCTGGTTTGCCCTGCTCCATGCGATGACGGCAACATAGGAGGAGAGGAGTTCTCGGTGACGCTGATGGCGAGGGAGAGGTGCGTGGGGAGGAAGAACAAAGGAATAACGTGGGGCTTCACTTCCATCATTGGGAGGCGGAGAGAGATGGAAGATGCCGTCGCCGTTATTCCTGGTTTTATGTCTTGCACATGCGATCATGTTGGCGGTTGTACGGCCTCCGGTTCTAGAACCTCCGGCGAGATCTCGCCCGTCCATTTCTTCGGCGTCTACGACGGTCATGGAGGCTCCCAGGTGGCCAAATTTTGTGCTGAGCGGATCCATGAAGTAGTAGCAGAGGAATGGGACAAGCTAGGAGGTGGTGGGGATGAGTGGCGTAAGAGGTGGGAAGCAGCATTTTATAGTGGTTTTGAGAGGGCTGACAATGAGGTGGTGACAGAAGCAATAGCGCCAGAAATGGTTGGATCAACTGCTATAGCAGTAGCTGTATCAGGGTGTCAGATTATTTCCTCCAACTGCGGTGACTCAAGGGCAGTGCTTTGTCGAGGCACTCAAGCCATCCCTTTAACTGTTGATCACAAGCCTGATAGAGAAGATGAACTGGTGAGGATTGAAGGAGGGGGAGGGAGAGTCATAAACTGGAATGGTGCTAGGGTATTTGGAGTACTTGCTATGTCCCGAGCTATAGGAGATCGGTATTTACGGCCATGGATCATTCCAGTACCTGAAGTTACTTTCATGACAAGAAGCGAAGATGATGAATGTTTGATAGTGGCAAGTGATGGGCTTTGGGATGTGATGTCCAATGATGAGGTCGGGGAGGTGGCTCGTCAAATATTAAGGCGGCGTCGCAGGTGTGCAACAGCTGATGGAAGTTCATCACCACCAGCACAAGCTGTTGCTGACCATCTTACTGACATAGCTTTCAGGAAAAACAGCTCTGATAACATTTCAGTCATTGTTGTCGATTTAAAAACAAAGAGGAAGCGCCAGCAGAGACAATGA

ncbi_104597107(PP2CA.1)

ATGGCGGAAATATGTTGCGGATTTGTTAGTGAAAGTGAAACAGCAGCGCCGTGCGAGCCAAATCCTCGAGCGGCAAGGCGCCGGAGGATGGAGATTCGACGGTTCAAGTTCATAGCGGGCGTGTCCATATCACAAGAGAATGGCCGGAAACGCCCAAGACTGGATGTTTTTACCGCCGTTTCGCCGCGGGATTGCGAGAACGCAGTTGAGAACTGTGGCATGAATGGGGTACAACAACATCGTTCGGCAGCGGAGGACGTTGAATCCGACACAAATTCTAACTCGAGTTCAGGTCGGTTATCGGTGCAGGAAATCTCAGGGCCGAGTGAGCCACCGGCTCCTGTTCTGGTCGAGCCGTTGTTCTTGGACCAACATCCTAAGTTCGGTATGACATCTGTTTGCGGCCGGAGAAGAGACATGGAAGATGCGGTAGCAATCCACCCTTCGTTTTTCCGTCGAGATCATCAGACTCCGGGGGGATTGCATTTTTTCGGCGTTTATGACGGCCATGGGTGCTCTCATGTGGCTAAGTCTTGTAAAGAACGGATGCATGAGGTGGTAAAGGAAGAGCTAGAAAACGGCTATGCATCTATCGAGTGGAAGGAGTCGATGGAGCGAAGCTTCTCTCGAATGGACAAGGAGGTACTAGTCGGTTACGAGGCGGTGTTGAATCCCAATTGCCGGTGCGAACTTCAGACGCCGCAGTGCGATGCCGTAGGATCAACAGCTGTCGTAGCCATAGTCACGCCGGAAAAAATTGTCGTCGCCAACTGCGGTGACTCCCGCGCCGTGCTGAGCCGCAACCGCAAAGCCGTCCCTCTCTCTACTGATCATAAGCCTGATCGACCAGATGAACTGCTTCGAATCCAAGCAGCTGGAGGCCGCGTAATATACTGGGACGGTCCACGGGTTCTCGGAGTTCTAGCTATGTCCAGAGCAATCGGAGACAATTACTTGAAGCCATACGTGATATCGGAACCGGAGGTAACGGTAACGGATCGGACGGACGAGGACGAGTGCCTAATTCTGGCAAGCGACGGACTGTGGGACGTGGTGTCGAGCGACACCGCGTGTAGCATAGCTCGCATGTGCCTGGGCGGGCAGGCACCGTCTCCGCCCGTGTCTCCGGGGAACGAAGTGAATGAAGGGGTGGCCGGAGAAAGCTCCGACAAGGCGTGCTCTGACGCGTCAATGTTGCTCACGAAGCTGGCCCTGGCGAGGAATAGTACGGACAACGTGAGCGTAGTCGTAATCGATCTCAGGCGTGACACGTAG

ncbi_104600371(PP2C51)

ATGTCGCCGGATTCCAGCAAGAGGGATGAATCTTTGTCGTTATCGTGTGTTAAGAGATCGTTGCATGTTCGCCGGAAAAGGCTAGAGAATCGACGTCTGAAAGCCGTCTCTTCCAGAGAGGACCTTGCGTCGCTCGGCGATGGTAACTCCGCCGACGTATCAAAGTTGGAAGATGATGCCAGGGTTTGTGTTGCTAAGAAGACACGATTGGCTAATAATGAAGTGGTGACGGTTTGTCAGTCGCGTAATGATGGTGATACGGAATTGAAAACCGATGCTTGTTCCGATGAGCTGCCGTCCAATCACTCGTCATTATCGTCGGCATCGTCGTCATCCTCGGAGAACGAAGGGGTACCGATGTTGGTCGGGGGCGGGGAAGTCTTCGGGCGAGGAGATAAATTGAGGATCCTTCCGTGTCTGTCGCACGGATCGGCTTCAGTGTGTGGGCGGCGAAGGGAGATGGAAGATGCAGTGACGGTGGTGCCGGGGTTTCTGAACGAGGAAACCGGGCTGTATGATTTTTTTGCCGTCTACGACGGCCACGGTGGTGCGAGGGTTGCTCAAGTGTGCCGGGAGCGGTTACACCGTCTACTGGCAAAGGAAATTGAAGGTAGGGAGGCGTCGGAGGTGGCGACGAAGAGGGAGGAGGATATTAAGTGGGAGGAGGTGATGTTGGCGTCCTTCGCAAAGATGGACGAAGAAATCAATGGTGATGAAGTGGTAGTGGAGGATGGATCGTCGTCCTTGAAGACGATCGGATCGACGGCTGTGGTGGCGCTAGTTGGGACGGAGAAAGTCGTCGTCGCCAATTGCGGCGACTCCAGAGCTGTTCTCTCCCGCGGCGGAGTAGCGATACCACTCTCCCGTGATCACAAGCCTGACAGGCCGGACGAAATGGAGAGAGTAGAAGCTGCAGGTGGAAGGGTGATAAACTGGAATGGATACCGCATCCTAGGAGTGCTTGCTACTTCAAGATCGATAGGGGATCAGTATCTGAAGCCATATGTGATATGTGAACCAGAGGTGACAGTGAGCAAGAGAACGGGGAGGGAAGAGTTTCTGATATTGGCTAGTGATGGGTTGTGGGATGTAGTGTCAAATGAGGTTGCATGCGAGGTGGTGAGAAGATGTCTGGATGGCCGGATAATGAGAAAATTGTCGGGGATGGGTGCAAGTGGAGCTGCTGAGGCTGCAGCGGTGCTAGCTGAATTGGCGATGGCTCGTGGGAGCAAAGACAACATTAGTGTTGTGGTTGTAGACCTCAGACCCCACCACGGATTAAATATTGGATCCATCTCTTGA

ncbi_104600643(PP2C16-like)

ATGGAGGAGATGTCTCCAGCGGTTGCAGTGCCATTTAGGTTAGGTAATACAATCTGTGATAATTCTGCTATTGCAACCCACATGGAAATTACAAGACTAAAATTATTAACGGACGCAGCAAGTCTTCTGTCGGATCCTGCTGCCAAGCACTCTTCTGAATCTGTTTGTAGCGGAGATGAAGGTTGCAATTGTAGTGATCAACGTACTGAAGTTGGTATTGCAACCATATCAGCACCTCAGCAGAACGGCAGGGAAGGAGCTACTGCATCGGTTGGGATGGAAATCCCGGATAATGAAAGCAACTGGGTTGGTACTGATGCTACAATTCATGATATCGAGGAAGATGATTCTCTTTCTGTGGGGGGCGACCAAGTATTGGATAGTTCTTGTTCTCTTTCTGTGGCTAGTGATACCAGTAGTCTATGTGGCGATGAAACATTGTCTTTTGATACAGCTTGTGAGTTAAGCACACCAAGTTCTGTGGTTGTTGAGAAGAACATCGAAAATGCTCATGTTATTGCAAAGTCCGCCACTTTGGGGGATTTAAATACTGACCAGGAGCCCATGGGTGAAATTCTTTCTGTGCCAGCGGGCTTTGAGGTTGAGATTCGAGAAGGGTTTGATCGAAAGGCATCGGCTGTGGTTCTTCAGTTGCCTCCAGAGGAAGGGATCTGCAGAACAGGAATGCGCAGTGTTTATGAATTGAGTTGTATACCTCTTTGGGGTAGTACATCTATCTGTGGAAGAAGACCAGAGATGGAAGATGCTGTTGCTACTGTACCTTGGTTTCTGAAAATTCCTATTAAAATGCTGACAGATGACCATGTTGTGGATGGTATGAACCAAAAGTTGATTCACTCAACTGCTCATTTCTTTGGAGTTTATGATGGCCATGGAGGCTCTCAGGTTGCTAATTACTGTCGTGAACGTATCCATTCGGCTTTGATTGAGGAGATAGAAACCTTGCAAGAAAGTATGGATGGTGGAAGTAATACTGATAATTGGAAACTGCAGTGGGAGAAAGCATTCACCAGTTGCTTTGTAAAAGTTGATGATGAGGTTGCAGGAAGAGTTGGCAGAGAAGGTGTAGAGGACAAGGATGATACCTCTGAACCTGGTCCTGAACCTATTGCACCAGAAACTGTTGGGTCTACAGCTGTGGTGGCTATTATTTGCTCGTCACACATCATAGTTGCAAATTGTGGTGATTCAAGAGCAGTCCTCTGTCGCGGCAAAGAGCCTATTCCATTGTCTGTTGATCATAAACCAAATAGGGAAGACGAGTATTCAAGGATTGAAGCAGCTGGAGGCAAGGTCATACAATGGAATGGATATCGTGTATTTGGTGTTCTTGCAATGTCAAGGTCCATTGGTGATAGATATTTGAAACCGTGCATCATTCCAGAACCAGAAGTCATGTTTATTCCTCGAGCAAAAGAAGACGAATGTCTGATTCTAGCCAGTGATGGTTTATGGGATGTTATGACAAATGAGGAGGTTTGTGAAGTGGCTCGAAGGCGAATCCTTCTCTGGCACAAAAAGAATGGTATCACCCTTCCTGCAGAAAGGGGTGATGGAGTTGATCCTGCTGCTCAATCAGCAGCTGAATTCCTCTCGAAGCTTGCTCTTCAGAAAGGTAGCAAGGACAACATTACCGTGGTTGTGGTGGATTTGAAAGCTCAGAGAAAGTTTAAGAGCAAAACCTGA

ncbi_104601109(PP2CA.2)

ATGGCGGAAATATGTTGTGGAGTTGTCAGTAAAAGCGAAACGGCGGCGCCGTGCGAGCCGAGCTCCCGAGCTGCCAGGCGCCGGAGGATGGAGATCCGGCGGTTTAAGTTTGTTGCTGGAGTTTCCATGCCACAGGAAAATTGCCGGAAACGGCCGAGATTGGGTGTTTATGGGGCTTTGTCGCCGCGGGATTGCGAAAACGCAGTTGAGAACTGTGGCATGAACGGAGTGCGAGAGAGTTTGGTCACGTCGGATGAGGAGTCAGATACGAATTCTAACTCCAATTCAGAACATTTGTCGGTTCAGGAAATCTCGGGACCGAGTGGATCTCCGGCTCCTGTTCTGGTTGAGCCGTTGGTGCTAGACCAACGTCCGAAGTTCGGTATGACATCTGTCTGCGGGCGTAGAAGGGACATGGAAGATGCGGTTGCAATTCATCCGTCATTTTGCCGGCGAGATGGTCATATTACGGCGGGATTACATTTTTTCGGCGTGTACGACGGCCACGGATGCTCCCATGTGGCCAAGTCTTGTAAAGATCGGCTGCATGTGCTGGTGAAGGAAGAGTTTGAAAACGGCAAGGCGTCTAGGGAGTGGAAAGAGACGATGGAGCGAAGCTTCTCTCGAATGGACAAGGAGGTGCTAGTAGGGAATGGACTATCGTGTCCGAGTCCGAACTGTCGATGCGAGCTTCAGACGCCGCAGTGCGATGCAGTTGGATCAACGGCTGTAGTTGCCGTAATCACGCCGGAAAAGATTGTAGTCTCCAACTGCGGCGACTCCCGCGCCGTACTATGCCGCAATGGCAAGGCTGTTCCTCTCTCGTCTGATCATAAGCCGGATCGACCGGATGAACTAATTCGAATACAAGCAGCTGGAGGGCGTGTAATATATTGGGACGGTCCACGGGTCCTTGGAGTTCTAGCCATGTCTAGGGCCATCGGGGACAATTACTTGAAGCCTTACGTGATATCGGAGCCGGAGGTGACGGTAACGGAGCGGACGGATGAAGATGACTGCCTAATACTGGCTAGCGACGGCCTGTGGGACGTGGTGTCGAACGAAACAGCATGCAGCATCGTTCGCATGTGCCTGCGCGGGCAGCCACTGCCGCCTCTGGTGTCTCCGGGGAACAATCTGAGTGACGGGGTTGCCGGAGAGAGCTCCGACAAGGCGTGTTCCGACGCGTCGATGTTGCTCACCAAGCTGGCCCTGGCCAGGCATAGTACGGACAATGTGAGCGTGGTCGTAATTAATCTGAGGCGCGACACGTAG

ncbi_104604491(PP2C06.2)

ATGGATCCGTTGGACGATGAGGATCTCCGTCAAGTCGGTTCTCGCCGGGGATCCGAAGTGGCATCGACGGATACCGGAGCATCTTCACTCTTGTCAACTGAAGACAGCCGGAGCATCACTGGCTCCAGCGATATCTCGGTCAGTAGCAGCAGCAGCTCCAGTGAGATTCCCACCGCAGTTCACGAGGATGCACTAGTGCCGAGGCTGGTAGGTACTAGGCCTAGCGTAGGAGGAGGAGAAGAGTTGACGACAACGCTGACGGTGAGGGAGAGGTGCGTGGGGAGGAACAACAAAGGAATGACGTGGGGATTCACTTCGATCATTGGGAGACGGAGAGAGATGGAGGATACCGTCGCCGTTATTCCAGGCTTTATGTCTCGAACCTGCGGTCTTATTGGAGGTTGTACGGCCCCCGGTTCTAGAAGCTCCGGCGAGATCTCGCCTGTCCATTTCTTCGGCGTCTACGACGGTCATGGAGGCTCTCAGGTGGCCAAATTTTGTGCTGAGCGGATCCATGAAGTAGTAGCAGAGGAATGGAACAAGATAGGAGGTGATGGAGATGGGTGGCGTAGGAGGTGGGAAGATGCATTTTGTATTGGTTTTGAGAGGGCTGACAATGAGGTGGTGACAGAATCAATAGCACCAGAAATAGTTGGATCCACTGCTGTAGCAGTGGCTGTATCTGGCTGTCAGATAATTACCTCCAATTGTGGTGACTCAAGAGCAGTGCTTTGTCGAGGGAGTCAAACAATCCCTTTAACTGTGGATCACAAGCCAGATAGAGATGATGAACTAGTGAGGATTGAAGGTGAGGGAGGGAGAGTTATAAACTGGAATGGTTCTAGAGTATTTGGAGTTCTTGCCATGTCCCGAGCTATAGGGGATAGGTATTTACGACCATGGATTATTCCAGTTCCTGAAGTTACTTTTACAACAAGAAGTGAAGATGACGAGTGTTTGATAGTGGCAAGTGATGGGCTTTGGGATGTGATGTCCAATGATGAGGTTGGGGAGATGGCTCGTCAAATTTTGAGGCGGCAACGCAGGCTCCCAATGGGTGACCGGAGTTCATCTTCACCGGCACAAGCTGTTGCTGACCATCTTACTGATGTAGCATACAGGAAAAACAGTTCTGACAACATCTCAGTCATTGTTGTGGACTTGAAATCAAAGAGGAAGCGTCAGCAAAGAGAATGA
